# A randomized, open-label study of the tolerability and efficacy of one or three daily doses of ivermectin plus diethylcarbamazine and albendazole (IDA) versus one dose of ivermectin plus albendazole (IA) for treatment of onchocerciasis

**DOI:** 10.1371/journal.pntd.0011365

**Published:** 2023-05-19

**Authors:** Nicholas O. Opoku, Felix Doe, Bettina Dubben, Nicole Fetcho, Kerstin Fischer, Peter U. Fischer, Shelter Gordor, Charles W. Goss, Michael E. Gyasi, Achim Hoerauf, Augustine R. Hong, Eric Kanza, Christopher L. King, Ruth Laryea, Daphne Lew, Mahmood A. Seidu, Gary J. Weil

**Affiliations:** 1 Fred Newton Binka School of Public Health, University of Health and Allied Sciences, Ho, Ghana; 2 Hohoe Municipal Hospital, Hohoe, Ghana; 3 Institute of Medical Microbiology, Immunology and Parasitology (IMMIP), University Hospital Bonn, Bonn, Germany; 4 Infectious Diseases Division, Department of Medicine, Washington University School of Medicine, St. Louis, Missouri, United States of America; 5 Division of Biostatistics, Washington University School of Medicine, St. Louis, Missouri; 6 St. Thomas Eye Hospital, Accra, Ghana; 7 Department of Ophthalmology, Washington University School of Medicine, St. Louis, Missouri, United States of America; 8 Centre de Recherche Clinique de Butembo, Université Catholique du Graben, Site Horizon, Butembo, Democratic Republic of the Congo (DRC); 9 Center for Global Health and Diseases, Case-Western Reserve University, Cleveland, Ohio, United States of America; 10 Department of Medical Laboratory Science, School of Biomedical and Allied Sciences, University of Ghana, Accra, Ghana; Erasmus MC, University Medical Center Rotterdam, NETHERLANDS

## Abstract

**Background:**

Onchocerciasis (“river blindness”) has been targeted for elimination. New treatments that kill or permanently sterilize female worms could accelerate this process. Prior studies have shown that triple drug treatment with ivermectin plus diethylcarbamazine and albendazole (IDA) leads to prolonged clearance of microfilaremia in persons with lymphatic filariasis. We now report results from a randomized clinical trial that compared the tolerability and efficacy of IDA vs. a comparator treatment (ivermectin plus albendazole, IA) in persons with onchocerciasis.

**Methods and findings:**

The study was performed in the Volta region of Ghana. Persons with microfiladermia and palpable subcutaneous nodules were pre-treated with two oral doses of ivermectin (150 μg/kg) separated by at least 6 months prior to treatment with either a single oral dose of ivermectin 150 μg/kg plus albendazole 400 mg (IA), a single oral dose of IDA (IDA1, IA plus diethylcarbamazine (DEC. 6 mg/kg) or three consecutive daily doses of IDA (IDA3). These treatments were tolerated equally well. While adverse events were common (approximately 30% overall), no severe or serious treatment-emergent adverse events were observed. Skin microfilariae were absent or present with very low densities after all three treatments through 18 months, at which time nodules were excised for histological assessment. Nodule histology was evaluated by two independent assessors who were masked regarding participant infection status or treatment assignment. Significantly lower percentages of female worms were alive and fertile in nodules recovered from study participants after IDA1 (40/261, 15.3%) and IDA3 (34/281, 12.1%) than after IA (41/180, 22.8%). This corresponds to a 40% reduction in the percentage of female worms that were alive and fertile after IDA treatments relative to results observed after the IA comparator treatment (P = 0.004). Percentages of female worms that were alive (a secondary outcome of the study) were also lower after IDA treatments (301/574, 52.4%) than after IA (127/198, 64.1%) (P = 0.004). Importantly, some comparisons (including the reduced % of fertile female worms after IDA1 vs IA treatment, which was the primary endpoint for the study) were not statistically significant when results were adjusted for intraclass correlation of worm fertility and viability for worms recovered from individual study participants.

**Conclusions:**

Results from this pilot study suggest that IDA was well tolerated after ivermectin pretreatment. They also suggest that IDA was more effective than the comparator treatment IA for killing or sterilizing female *O*. *volvulus* worms. No other short-course oral treatment for onchocerciasis has been demonstrated to have macrofilaricidal activity. However, this first study was too small to provide conclusive results. Therefore, additional studies will be needed to confirm these promising findings.

**Trial registration:**

The study is registered at Cinicaltrials.gov under the number NCT04188301.

## Author summary

**Why was this study done?:**

- Onchocerciasis is a neglected tropical disease caused by parasitic round worms. The infective worm larvae are transmitted by black flies that breed near rivers in sub-Saharan Africa. The parasites cause severe skin disease with itching and ocular disease; the common name for the infection is “river blindness”. WHO has targeted onchocerciasis for elimination and mass drug administration is the key intervention strategy.
- There is an urgent need for improved treatments to kill or permanently sterilize the adult parasites. This study tested a combination drug treatment that is quite effective for treating other related worm infections.

**What did the researchers do and find?:**

- We performed a randomized clinical trial that compared a triple drug combination with a standard of care two-drug treatment that is widely used in Africa. We found that the new treatment was as safe as the standard treatment.
- More importantly, analysis of adult female worms surgically removed from study participants suggested that the new treatment might be more effective than the standard treatment for killing or permanently sterilizing the parasites. However, this first study was too small to provide conclusive results.

**What do these findings mean?:**

- An improved treatment could improve chances for eliminating onchocerciasis in Africa.
- Results from this study can be used to better plan follow-up studies to explore the value of this and other new combination treatments for onchocerciasis.

## Introduction

Onchocerciasis (human infection with the filarial nematode *Onchocerca volvulus*) is transmitted by biting *Simulium* blackflies that breed in rivers. Larval parasites (microfilariae, Mf) live in the skin and sometimes migrate to the eyes. Host inflammatory responses to Mf can lead to severe dermatitis and ocular disease (“river blindness”). The World Health Organization (WHO) estimates that at least 25 million people are infected in 31 countries in sub-Saharan Africa [1,2]. An estimated one million people have severe visual impairment or blindness due to onchocerciasis, and it is the world’s second leading infectious cause of blindness [1,2]. The drug ivermectin (IVM) clears Mf from the skin and eyes and temporarily sterilizes adult female *O*. *volvulus* worms that live in subcutaneous nodules. Mass drug administration (MDA) of IVM has dramatically reduced the prevalence and intensity of *O*. *volvulus* infections in Africa so that severe ocular disease is now uncommon in most endemic areas [1,3]. However, IVM has no permanent effect on adult *O*. *volvulus* worms that can live for up to 15 years. Thus, IVM has only been successful for interrupting onchocerciasis transmission in areas with low transmission, and/or in geographically isolated areas such as in the Latin America [1,4], Senegal and Mali [5] or Northern Sudan [6]. In contrast, repeated rounds of MDA with IVM have not been sufficient to interrupt transmission in many other areas [1]. There is an urgent need to find new drugs or novel combinations of existing drugs that can kill or permanently sterilize adult worms [7,8]. Such treatments could accelerate elimination of the infection in Africa.

Recent studies have shown that IDA [a single co-administered dose of IVM with diethylcarbamazine (DEC) and albendazole (ALB)] is superior to legacy two-drug treatments (IVM plus ALB or DEC plus ALB) for clearing Mf from the blood of humans with lymphatic filariasis (LF, which is caused by nematodes related to *O*. *volvulus*) [9–14]. IDA has partial macrofilaridal activity against *Wuchereria bancrofti*, and it also appears to permanently sterilize some adult worms that survive the treatment [15]. Although adult *O*. *volvulus* worms are less susceptible to treatment than other filarial species, we hypothesized that IDA might also be effective for killing or permanently sterilizing *O*. *volvulus* adult worms. The WHO does not currently recommend use of IDA for LF elimination in areas that are coendemic for LF and onchocerciasis [16]. That is because repeated doses of DEC can cause severe/serious ocular adverse events in persons with high density ocular *O*. *volvulus* infections. Because IVM clears or greatly reduces Mf in the skin and eyes of persons with onchocerciasis [17,18], we hypothesized that IVM pretreatment would make it safe for people with onchocerciasis to be treated with IDA. Thus, the purpose of this study was to assess the safety and efficacy of IDA treatment in onchocerciasis patients after IVM pretreatment.

## Methods

### Ethics statement

The protocol was reviewed and approved by ethical review committees at the University of Health and Allied Sciences (UHAS) in Ho, Ghana, the Ghana Health Service, The Ghana Food and Drug Administration, Case-Western Reserve University (Cleveland, OH) and Washington University School of Medicine (St. Louis, MO). Safety data were reviewed periodically by an independent, project-specific data safety monitoring board (DSMB).

### Protocol

This was a randomized, parallel-group, open-label clinical trial. The purpose of the study was to provide the first data on the safety and efficacy of IDA treatment of onchocerciasis relative to treatment with the comparator regimen of IVM plus ALB. All treatments were provided after pre-treatment with IVM to reduce Mf counts in the skin and eyes. The primary safety objective for the study was to compare rates and types of severe adverse events that occur within seven days after different treatments. The primary efficacy objective of the study was to compare the effect of different treatments for killing or sterilizing adult female *O*. *volvulus* worms. Secondary endpoints included (among others) adult worm killing and complete clearance of Mf from the skin 18 months after treatment. The full study protocol is provided as supplemental information **S1 Protocol**.

### Inclusion and exclusion criteria

This study (Part 2) enrolled people with onchocerciasis who had participated in an earlier (Part 1) study of the efficacy of IVM for clearing *O*. *volvulus* Mf from the skin and eyes. The protocol, safety and efficacy results from the Part 1 study have been published [19]. Briefly, that study enrolled persons between the ages of 16 and 70 with at least one palpable subcutaneous nodule (onchocercoma) and ≥ 1 Mf/mg of skin. The geometric mean skin snip Mf count for participants in the Part 1 study was 12.7/mg (range 3–86), and 27.7% of participants had Mf visible in the anterior chamber (MfAC) in one or both eyes before IVM treatment. Inclusion criteria for the present (Part 2) study included participation in the Part 1 study with a baseline skin Mf count of ≥ 3 mg/mg prior to the Part 1 IVM treatment. Exclusion criteria for the Part 2 study included severe ocular disease in either eye (e.g., uveitis, severe glaucoma, severe keratitis, and/or cataracts that interfere with visualization of the posterior segment of the eye. Please see the protocol for details). In addition, persons with > 5 MfAC in either eye or with one or more Mf detected in the posterior segment of either eye at the time of enrollment (post-IVM) were to be excluded. These ocular exclusion criteria were added to avoid confusion between baseline ocular abnormalities and treatment emergent adverse events (TEAE). We also wanted to minimize the risk of IDA exacerbating pre-existing ocular disease in this first use of IDA for treatment of onchocerciasis. Other exclusion criteria were pregnancy, known allergy to study medications, significant comorbidities such as renal insufficiency, severe hepatitis, or other acute or chronic illnesses that interfered with the participant’s ability to work or perform routine household chores.

### Screening and participant enrollment

Screening and recruitment were performed in Nkwanta North District in the Volta region of Ghana (approximately 4 hours by car from the clinical trial center in Hohoe, Ghana). Screening was conducted in communities that are hypoendemic for onchocerciasis (nodule prevalence < 20%) where MDA of IVM had only recently been implemented. The study team met with community leaders and local health personnel in Nkwanta North and held open community meetings to explain the purposes and plans for the study prior to screening and recruitment of participants. The meetings and consent forms were in English and a local language (either Twi or Konkomba) used in the study area. Participation required written consent for adults and written consent from a parent or guardian plus assent for minors younger than 18 years of age.

### Medical history and physical examinations

A brief medical history reviewed prior illnesses and current medications. This included an oral review of systems to identify baseline symptoms with special attention to any history of prior onchocercal eye or skin disease or treatment. Serum tests for aspartate aminotransferase, alanine transaminase, and creatinine were performed to rule out serious liver or kidney disease. The physical examination included height, weight, and vital signs with special attention to skin lesions and lymph nodes. Onchocercal nodules were detected by manual palpation.

### Skin snip examinations to detect Mf

Four skin snips were collected (one from each posterior iliac crest and posterior calf) with a Holth corneoscleral punch (Everhards, Meckenheim, Germany). Snips were weighed and incubated in 100 μl of isotonic saline in individual wells of a flat-bottomed microtiter plate at ambient temperature for at least 8 hours. Snips in the microtiter wells were then examined with an inverted microscope, and Mf were counted by experienced microscopists. Mean values for Mf/mg for four snips were calculated. Persons who performed skin snips or counted Mf by microscopy were masked with respect to participant treatments.

### Ophthalmological examinations

A panel of tests was performed as described in detail in the study protocol. Briefly, the panel included tests of visual acuity, color vision, visual field testing by frequency doubling technology (FDT) perimetry, pupillary reflex, applanation tonometry, indirect ophthalmoscopy, fundus photography and optical coherence tomography (OCT), which provides detailed images of the posterior segment including the retina. Slit lamp examinations were performed to assess ocular abnormalities in the cornea and anterior segment. Participants sat with their heads bent as far forward and down for 10 minutes as tolerated prior to the slit lamp examination to optimize visualization of Mf in the anterior chamber. The total ocular Mf count was calculated by summing the numbers of Mf identified in the anterior chamber of each eye.

### Drug treatment, adverse event (AE) assessments, and follow-up

Participants were pretreated with IVM 150 μg/kg by mouth at least 6 months before the planned Part 2 study treatment to clear or reduce Mf counts in skin and eyes. A second IVM pretreatment was provided to all participants because of delayed regulatory approval for the Part 2 study and delays related to SARS-2-COVID lockdowns. The second IVM pretreatments were provided more than 1 year after the first pretreatment. The median interval between the second IVM pretreatment and the Part 2 study treatment was 7.3 weeks (range 1–28 weeks).

Participants were transported from their home villages to the UHAS School of Public Health Research Centre (which is located within the grounds of the Hohoe Municipal Hospital) for Part 2 treatments and clinical evaluations. A study statistician prepared a random treatment allocation schedule and participants were randomized sequentially into one of the three treatment arms by the study pharmacist. The arms included a single dose of IVM 150 μg/kg plus ALB 400 mg fixed dose (IA), a single dose of IA plus DEC 6 mg/kg (IDA1), or three consecutive daily doses of IDA (IDA3). All treatments were oral and directly observed. Participants were evaluated daily for 7 days after treatment and asked whether they had symptoms suggestive of systemic (e.g., fever, headache), cutaneous, or ocular AEs. A study physician performed a directed physical examination for all participants. All participants had full ophthalmological examinations as described above on the day before treatment, on days 3 and 7 after treatment, and 3 months after treatment. A skin snip test was performed shortly before treatment, and this was repeated at 12 and 18 months after treatment.

### Treatment masking

While this was an open-label study, medical/technical staff who assessed skin Mf, AEs, performed eye examinations, and assessed nodule histology were masked with regard to treatment arm. The study pharmacist and his assistant were responsible for treating study participants.

### Data acquisition, transfer, and management

The study used an electronic data capture (EDC) system developed by CliniOps (Fremont, CA, USA) to capture and transfer clinical data. De-identified clinical data were entered directly into tablet computers loaded with a mobile data management application called CliniTrial. The data were entered by designated, trained members of the UHAS research team on the day of enrollment or AE assessment. A parallel participant key (separate from CliniTrial and maintained at UHAS) linked study ID numbers with personal identifying information such as name and date of birth. The participant key was not shared with investigators or staff at Washington University.

The EDC system employed is 21 CFR Part 11 compliant, and electronic case report forms (CRFs) were developed to comply with International Council for Harmonization on Good Clinical Practice (ICH GCP) and CDASH/CDISC standards [20]. Validation checks and automated alert checks were programmed into the EDC system to maintain a high level of data quality at the point of entry. Data were entered into tablet computers, and encrypted data were uploaded daily to a secured cloud server via the internet. The cloud server uses multi-available zone and geo-redundant backups to protect against data loss. A data manager at Washington University performed additional data cleaning and validation and communicated with the UHAS data manager and study investigators to clear queries prior to data lock. AEs were coded using MedDRA dictionaries (version 20.0) [21]. Paper case report forms were used for backup in case of EDC or equipment malfunction and for documentation of serious adverse events. All written forms (i.e., consent and backup data collection forms) were stored at the study site per Ghana FDA requirements for storing source documents.

Laboratory test results (skin snip Mf counts, blood analysis data etc.) were recorded on paper forms and transferred into REDCap software (https://projectredcap.org) for analysis.

### AE assessment

Adverse events were scored using a modified version of the National Cancer Institute Common Terminology Criteria for Adverse Events tables, version 4.0. Study ophthalmologists added additional details regarding ocular AEs to the CTCAE tables for this study. The tables were used to classify and score the severity of adverse events. Briefly, grade 1 AEs are mild events that would not prevent participants from working or performing household chores. Grade 2 AEs are moderate events that would prevent work or performance of household chores. Records of participants with AEs with severity of grade 3 or higher that interfered with activities of daily living were to be evaluated by an independent medical monitor who was not part of the research team. The medical monitor’s role was to review the report with the lead study physician to determine whether it met criteria for a serious adverse event (SAE) and whether the AE was related to the study treatment [22].

### Nodulectomy and processing of nodules

Surgical removal of onchocercomas was performed at the Hohoe Municipal Hospital 17.5 to 18.5 months after treatment using standard procedures [23]. Small nodules (≤1 cm in diameter) were placed in 80% ethanol. Larger nodules (>1 cm in diameter) were cut in half prior to fixation. Nodule samples were transported to the Pathology Department at the University of Ghana, School of Medicine in Accra and embedded in paraffin as described previously [24]. Each paraffin block received a unique barcode that was linked in a separate database with other participant information. Technicians working on the nodules did not know treatment histories or whether different nodules belonged to the same participant. Paraffin blocks were shipped to Washington University in St. Louis for further processing. Ten consecutive 5 μm sections were cut from each block with a rotary microtome HM340E (MICROM, Laborgeräte GmbH, Jena, Germany). Blocks that could not be cut because of calcification were examined with a dissecting microscope to detect calcified worm structures.

### Histology, digitalization and evaluation of nodules

Two consecutive sections were stained with Meyer’s hematoxylin & eosin (H&E, Merck, Darmstadt, Germany), and a polyclonal rabbit antibody directed against an *O*. *volvulus* aspartic protease (APR, GenBank U81605) for assessment of worm viability [25]. Stained slides were scanned using an Olympus scanner (Olympus VS120 Brightfield Slide Scanning System, Tokyo, Japan) at 20X magnification [26]. The digital images were checked for quality and uploaded to a secure cloud server for assessment. The digital images of nodule sections (mean file size ~9.5 GB) were assessed independently by two readers using the open source viewing software OlyVia 2.19 (Olympus). Each reader recorded results using a digital case report form in REDCap. One reader (BD) was based in Bonn, Germany and the other reader (KF) was based in St. Louis, USA. Images were read in the same orientation relative to barcode labels on the scanned slides to facilitate communication and comparisons. The readers reviewed nodule sections, counted worms, and judged the viability and fertility of adult *O*. *volvulus* worms according to previously published criteria [27,28]. Females with collapsed uterus branches were not included in the fertility analysis. Females with morulae or later stage embryos in the uterus were considered to be fertile (Fig 2G). Dead worms were recorded as females unless they were clearly identifiable as males. Heavily calcified nodules (Fig 2A) that could not be sectioned were arbitrarily (and conservatively) considered to contain one dead female worm. The nodule assessments of both readers were compared using SAS software (SAS Institute Inc., Cary, NC, USA, https://www.sas.com). After initial readings were completed, readers met virtually to resolve discrepancies and finalize results before the code was broken.

**Fig 1:**
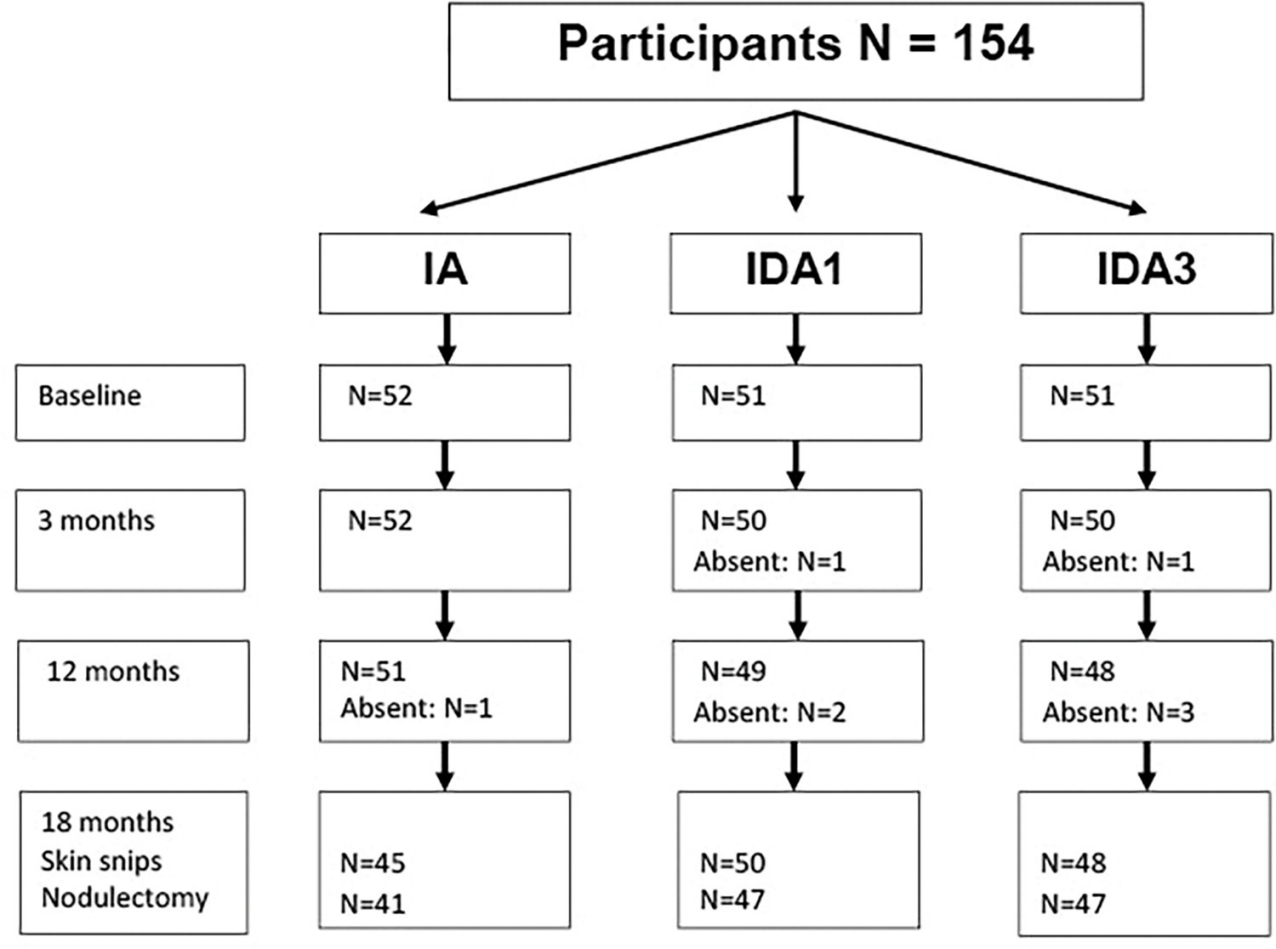
CONSORT diagram for the Part 2 study. The diagram shows the number of persons treated at baseline and evaluated at each follow-up time point for adverse event assessment, skin snip testing, and nodulectomy (18 months only). Eight participants refused nodulectomy.

**Fig 2:**
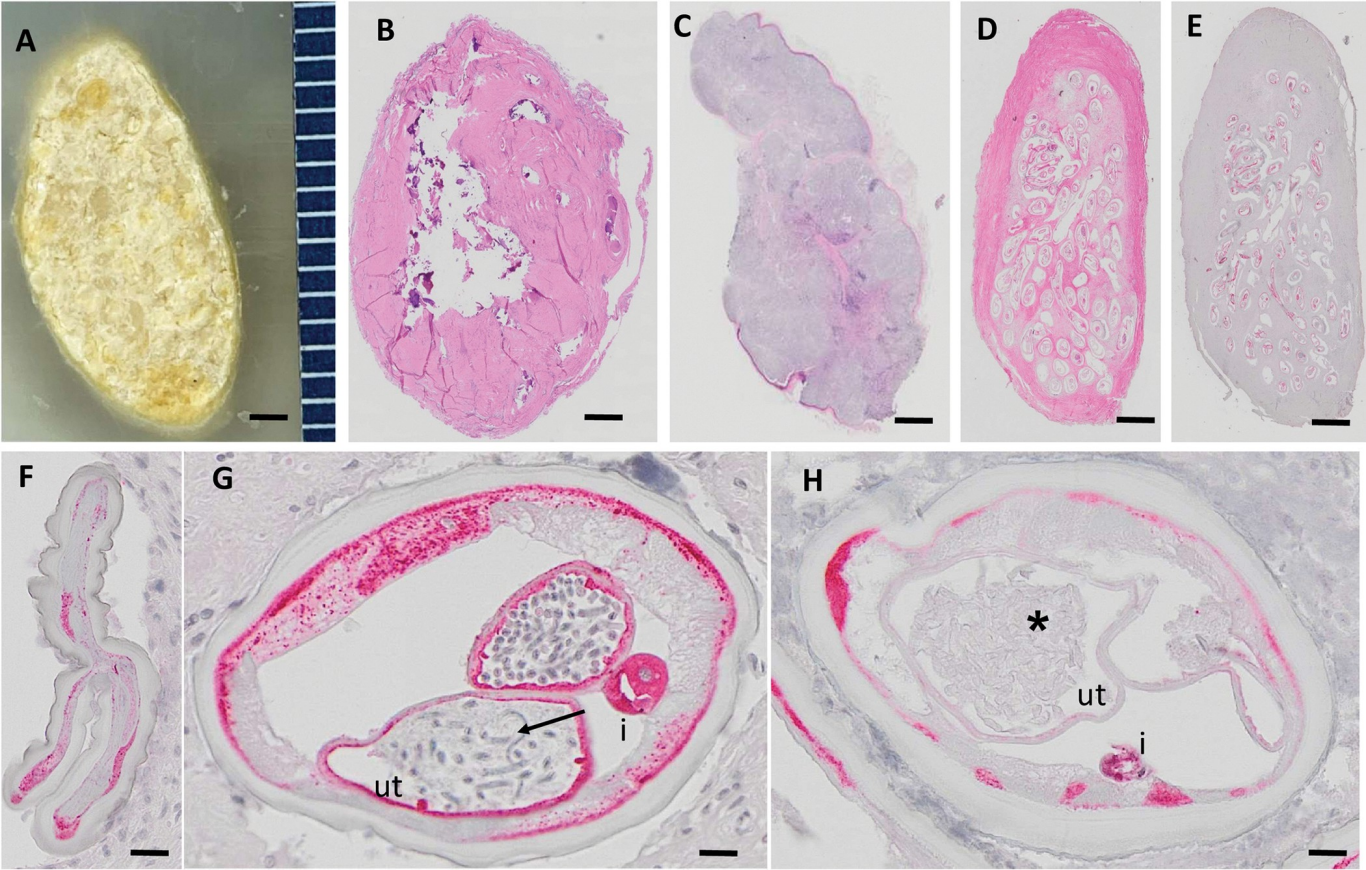
Examples of nodules and worm sections analyzed by histology. Panels B-D are H&E stained sections, E-H are APR stained sections; red staining indicates that the worms are alive. A: Paraffin block with a highly calcified nodule from an IDA1 treated participant that could not be sectioned and was classified as “not able to cut, one dead female”. B: Nodule with calcified (dead) worms from an IDA3 treated participant. C: An excised nodule that was not evaluated because it was a lymph node. D-E: Consecutive sections from an IDA3 nodule with two living female worms stained with H&E and APR, respectively. F: This nodule contains a living *O*. *volvulus* female that could not be evaluated for embryogenesis, because the uteri were collapsed. G-H: Cross-sections of two different living *O*. *volvulus* females in the same nodule from a person who had been treated with IDA3. Image G shows normal stretched microfilariae in the uterus (arrow). In contrast, the worm uterus in image H is filled with degenerated embryos (asterisk). Ut, uterus; I, intestine. Scale bars: A-E 1 mm; F-H 20 μm.

### Data management for skin snip and biochemistry laboratory results

Data were recorded on paper case report forms and later entered into REDCap at the UHAS School of Public Health; that data center also validated and cleaned the data. REDCap files included participants’ study identification numbers without personal identifiers. Encrypted REDCap data were transferred to a dedicated server housed at Washington University in St. Louis. A data manager at Washington University performed additional data cleaning and validation and communicated with the UHAS data manager and study investigators to clear queries before data lock.

### Statistical methods

Descriptive statistics were calculated as frequencies and proportions for categorical variables; arithmetic means ± standard deviation (SD) and geometric means with 95% CIs were calculated for continuous variables. Comparisons between treatment groups for demographic, infection, and adverse-event variables were performed using chi-square and Fisher’s exact tests (as appropriate) for categorical variables; analysis of variance (ANOVA) was used for continuous variables, and data were log transformed as needed to meet model assumptions. Analysis of the primary (% fertile female worms) and secondary (% living female worms) efficacy outcomes was conducted using a mixed-effects logistic regression model where study participant was included as a random effect to adjust for multiple worms per person. The covariates age, sex, and Mf density at baseline were also evaluated for inclusion in these models, and they were retained if they were significant (P < 0.05). All analyses were conducted in SAS for Windows version 9.4 (SAS Institute), and P < 0.05 was used to determine statistical significance.

## Results

### Enrollment and demographics by treatment group

The CONSORT diagram for the Part 1 study (S1 Fig) starts with screening performed prior to IVM pretreatment) and describes how participants in the Part 1 study were selected or excluded for the Part 2 study. Participant enrollment in Part 2 and follow-up at different time points are summarized in a second CONSORT diagram (Fig 1). Forty-two people who were pretreated with IVM in Part 1 were excluded from the Part 2 study based on abnormalities detected during detailed ophthalmological examinations conducted during the Part 1 IVM pre-treatment study. This included 8 people who were excluded based on rescreening performed just prior to Part 2. The most common exclusionary conditions were glaucoma (16), retinal scar (11), cataract preventing adequate fundus photography of the retina (8), and macular drusen (8). Persons with conditions that required treatment or follow-up were referred to Ghana Health Service facilities for care. A full description of ophthalmological findings prior to the Part 2 study will be published separately, as that is beyond the scope of this paper.

All Part 2 study participants had participated in the Part 1 study and received an additional IVM pretreatment as described in Methods. Table 1 shows baseline characteristics for persons enrolled in the study along with *O*. *volvulus* infection parameters prior to pretreatment with IVM and just prior to treatment in this study, by treatment group. A total of 154 persons were randomized and treated with Part 2 study medications. Four participants (2.6%) had MfAC and 13 (8.4%) had Mf-positive skin snips just prior to treatment (Table 1). The three treatment groups were generally comparable, but no participant in the IDA3 group had Mf detected in skin snips taken just before treatment; that group was also slightly younger than the other treatment groups. These differences occurred by chance during randomization. The male predominance in the study is due to the fact that men were more willing than women to participate in this study which required 9 or 10 days of testing, treatment and observation in a clinical trial facility that was located four hours away from their home villages.

**Table 1:** Demographic and *O*. *volvulus* infection parameters prior to ivermectin pre-treatment and just prior to the Part 2 treatment* by treatment group.

Variable	Group	IA (N = 52)	IDA1 (N = 51)	IDA3 (N = 51)	*P*-value
**Gender**	Female	21 (40.4%)	18 (35.3%)	11 (21.6%)	0.109
	Male	31 (59.6%)	33 (64.7%)	40 (78.4%)	
**Age (years)**		40.3 +/- 12.18	38.52 +/- 14.08	34.08 +/- 13.14	0.017
**BMI (kg/m** ^**2**^ **)**		20.23 +/- 2.3	20.12 +/- 2.02	20.45 +/- 2.19	0.609
**Before first ivermectin pre-treatment**					
**Mf density in skin snips**	Mf/mg**	12 (8.7, 16.4)	10.8 (8.4, 13.9)	10.9 (8.3, 14.3)	0.854
**Mf in the anterior chamber of the eye**	No	44 (84.6%)	38 (74.5%)	39 (76.5%)	0.291
	Yes	8 (15.4%)	13 (25.5%)	14 (27.5%)	
	Mf/AC	2.8 (1.2, 7.0)	2.9 (1.6, 5.1)	2.2 (1.3, 3.7)	0.743
**Just prior to the Part 2 treatment**					
**Mf present in skin snips**	No	45 (86.5%)	45 (88.2%)	51 (100%)	0.014
	Yes	7 (13.5%)	6 (11.8%)	0 (0%)	
	Mf/mg**	0.57 (0.03, 0.98)	0.34 (0.18, 0.65)	0	0.149
**Mf in the anterior chamber of the eye**	No	50 (96.2%)	49 (96.1%)	51 (100%)	0.547
	Yes	2 (3.8%)	2 (3.9%)	(0%)	

*Treatments were IVM plus ALB (IA), one dose of IVM plus DEC and ALB (IDA1), and three doses of IVM plus DEC and ALB (IDA3).

** Mf (microfilaria) densities were calculated for Mf-positive subjects as geometric means (95% CI). Statistical comparison of Mf densities only considered IA and IDA1 treatment groups, because no Mf positives in the IDA3 group.

### Adverse events post-treatment

The frequency and severity of AEs recorded during the 7 day inpatient post-treatment observation period are summarized by AE type and treatment group in Table 2. A total of 101 AEs were recorded in 49 study participants. The most common non-ocular AEs were itching skin (6.5%), muscle/joint pain (3.9%), diarrhea (3.9%), chills and headache (3.2% for each). There were no significant differences in the frequency, severity, or type of AEs by treatment group. No severe or serious treatment-emergent AEs were reported; 91% of all AEs were grade 1, and 9% were grade 2 (mostly Mazzotti reactions, defined on page 29 of S1 Protocol). More details regarding ocular AEs are provided below.

**Table 2:** Post-treatment adverse events (AEs) by category and treatment group that were recorded within 7 days of treatment.

AE Type	Treatment arm*	Individuals reporting AEs	P-value
**Any AE**	IA	16/52 (30.8%)	0.557
	IDA1	19/51 (37.3%)	
	IDA3	14/51 (27.5%)	
**Ocular AE**	IA	4/52 (7.7%)	>0.999
	IDA1	4/51 (7.8%)	
	IDA3	3/51 (5.9%)	

*Treatments were IVM plus ALB (IA), one does of IVM plus DEC and ALB (IDA1), and three doses of IVM plus DEC and ALB, (IDA3).

Two deaths (serious adverse events) occurred in study participants long after they were treated in this study. The first case was a 29 year old man who died with typhoid fever 13 months after he was treated with IDA. The second case was a 42 year old man who died with an acute abdomen (despite laparatomy) 11 months after he was treated with IDA. Both of these cases were considered by the PI of the study and the Medical Monitor to have been unrelated to the study treatment.

### Ocular findings and treatment-emergent ocular adverse events

Only six of 154 study participants had MfAC detected at any time during the study (Part 2). These included participants with MfAC at baseline only with total MfAC counts from both eyes of 1, 1, and 8, one with MfAC at baseline and at 3 months after treatment (total MfAC was 1 at both time points), one with MfAC (1 Mf seen) on day 7 after treatment, and one with MfAC (1 Mf) only at 3 months after treatment. Ocular adverse events were documented in 16 participants within three months after treatment, and these were equally distributed across the three treatment groups (see **S1 Table**).

All ocular adverse events were of mild or moderate severity. No participant with MfAC observed at any time during the Part 2 study experienced an ocular AE. Intraocular AEs included vitritis, worsening cataract, retinal vascular disorders, and punctate corneal opacity. Three participants developed mild vitritis that did not affect visual acuity and spontaneously resolved later in the study. These participants did not report floaters, and they did not have anterior uveitis. Retinal vascular AEs (2) included a cotton wool spot with onset 3 months after treatment in one participant and a retinal flame hemorrhage in one participant that was first noticed on day 3 after treatment and resolved spontaneously. Two participants had reduced pinhole visual acuity after treatment compared to baseline. One had itching and mild worsening of a baseline cataract in the right eye, and the other had worsening of cataracts in both eyes. Sixteen of 21 (76.2%) of all ocular AEs had resolved by the 3 month follow-up examination. Persistent AEs included mild worsening cataracts (2 subjects in the IA arm, 1 subject in the IDA3 arm), punctate opacity in the cornea that was believed to be related to a small scar related to particulate (sand) injury (1 subject in the IDA3 arm), and the cotton wool spot which was first seen at 3 months and resolved later (1 subject in the IDA3 arm). None of the late AEs were associated with reduced visual acuity.

Optical coherence tomography imaging was performed prior to treatment and on day 3, day 7 and month 3 after treatment. Two parameters commonly assessed by OCT are central subfield thickness of the macula (CST) and optic nerve retinal nerve fiber layer (RNFL) thickness. No changes in CST or RNFL were observed after treatment. 144 participants had no change in visual field interpretation at any time during the study. Also, no participant with a normal baseline FDT (visual field) test had an abnormal result at the time of their final FTD test. Four participants with normal baseline FDT examinations had abnormal results on day 3 or 7 after treatment. However, FDT scores were normal in all of these participants by 3 months after treatment. Six participants with abnormal visual fields at baseline had normal visual field results during subsequent visits. Thus, the few differences noted in FDT results during this study were likely due to day to day variability in the assessment, and we do not consider them to represent ocular adverse events related to treatment.

### Effects of treatment on microfiladermia

Skin Mf data by treatment group over time are presented in Table 3. Mf counts were very low at baseline as expected after IVM pretreatment, and they remained very low through 3 months after the study treatments. Many participants had microfiladermia at 12 and 18 months; mean Mf counts did not differ by treatment group at those time points. Although there was a trend toward reduced Mf prevalence at 18 months in persons treated with IDA (single dose or 3 daily doses) compared to those treated with IA, the difference was not statistically significant (*P* = 0.09).

**Table 3:** Skin microfilaria prevalence and densities (Mf/mg) before and after treatment.

	Treatment	Baseline	Month3	Month12	Month18
Prevalence*	IA	7 (13.5%)	8 (15.4%)	23 (46%)	20 (66.7%)
	IDA1	6 (11.8%)	1 (2%)	21 (42.9%)	15 (44.1%)
	IDA3	0 (0%)	4 (8%)	21 (43.8%)	17 (53.1%)
Mf Count, Geometric Mean (95%CI)	IA	3.1 (1.8, 5.5)	1.9 (1, 3.4)	4.9 (2.8, 8.5)	7 (3.6, 13.9)
	IDA1	1.7 (0.8, 3.6)	3**	2.5 (1.6, 3.8)	9 (4.6, 17.6)
	IDA3	NA***	2.1 (0.9, 4.7)	4.7 (2.8, 7.9)	8.6 (4.3, 17.1)
Mf Count, Median, (Min, Max)	IA	0, (0, 9)	0, (0, 6)	0, (0, 41)	2, (0, 51)
	IDA1	0, (0, 6)	0, (0, 3)	0, (0, 23)	0, (0, 60)
	IDA3	0, (0, 0)	0, (0, 3)	0, (0, 36)	1, (0, 84)

* Treatments were IVM plus ALB (IA), one dose of IVM plus DEC and ALB (IDA1), and three doses of IVM plus DEC and ALB (IDA3). Microfilaria (Mf).

** 95% CI could not be estimated because raw data had only one nonzero value

*** Geometric mean could not be estimated because all values were zero

### Assessment of adult worms in onchocercal nodules

134 subjects underwent nodulectomy (89% of all persons enrolled in the study), and a total of 450 nodules containing 772 female worms were recovered (Table 4). Representative nodule histology images are shown in Fig 2. Not all nodules could be processed or evaluated, and not all female worms seen in nodules could be assessed for embryogenesis. For example, 18 nodules could not be evaluated (three after IA, six after IDA1, and 9 after IDA3). In some cases, this was because the excised material was not an onchocercoma (e.g., a lymphnode). In other cases, this was because no worms were visible in histologic sections. These “nodules” were not included in the efficacy analysis.

**Table 4:** Nodule and adult worm data by treatment group*.

	IA	IDA1	IDA3)	Total
Number of participants with nodulectomy (%)	41 (78.8)	46** (90.2)	47 (92.2)	134
Number of nodules (evaluable or not)	119	155	176	450
Median (IQR) nodules per participant	2 (2,4)	3 (2,4)	3, (2,5)	
Number of nodules (evaluated)	116	149	167	432
Number nodules with Mf (%)	19 (16.4)	24 (16.1)	19 (11.4)	62
Number of nodules with female worms (%)	111 (95.7)	148 (99.3)	166 (99.4)	425
Total number of female worms	198	274	300	772
Median (IQR) female worms per participant	4 (2,7)	5 (2.9)	5 (3,9)	
Number of living female worms (%)	127 (64.1)	142 (51.8)	159 (53)	428
Number of worms evaluated for fertility***	180	261	281	722
Number of fertile female worms* (%)	41 (22.8%)	40 (15.3%)	34 (12.1%)	115
Number of male worms	43	53	49	145

*Treatments were a single dose of IVM plus ALB (IA), a single dose of IVM plus DEC and ALB (IDA1), or three daily doses of IVM plus DEC and ALB (IDA3).

**One participant in the IDA1 treatment group who is listed as having nodulectomy in Fig 1 had only one nodule excised that contained a single worm that could not be evaluated. For that reason, this person is excluded from the first row in this table.

***These numbers exclude living female worms with collapsed uteri that could not be evaluated for fertility.

### Calcification

Twenty-nine nodules could not be sectioned because of heavy calcification (Fig 2A). These nodules were macroscopically examined and conservatively assumed to contain one dead adult female worm. The frequencies of such heavily calcified nodules were 6/116 (5.2%), 16/149 (10.7%) and 7/167 (6%) for onchocercal nodules in persons who had been treated with IA, IDA1, or IDA3, respectively (differences not statistically significant). More recovered nodules contained calcified dead or moribund adult female *O*. *volvulus* after IDA1 (55/148, 37.2%) or IDA3 (58/166, 34.9%) than after IA (28/111, 25.3%) (P = 0.024 for results after IDA1 or IDA3 vs IA).

### Female worm fertility assessment

The data are summarized in Table 4. Embryogenesis could not be evaluated for 49 of 428 female worms (11.4% overall, which included 17, 12, and 20 female worms with collapsed uteri in nodules from persons treated with IA, IDA1, and IDA3, respectively, see Fig 2F and S2 Table). Abnormal embryos were easily identified in other worms by the presence of tissue debris and disintegrating nuclei within the uterus (Fig 2H). Evaluable female worms in nodules from IA treated participants included 41/180 (22.8%) that were fertile (with morulae or later stage embryos in the uterus) (Fig 2G and S2 Table). In contrast, only 40 of 274 (15.3%) females in nodules from the IDA1 group and 34/300 (11.3%) of females in nodules from the IDA3 group had normal embryogenesis. Thus fertile worms divided by the total number of evaluable worms in nodules recovered from participants treated with IDA (13.7% for combined data for IDA1 and IDA3) was 40% lower than the percentage of fertile females in nodules recovered from persons who had received the comparator treatment (IA); this difference was statistically significant by Chi square (P = 0.004). Percentages of female worms that were alive (a secondary outcome in the study) were also lower after IDA treatments (301/574 or 52.4%) than after IA (127/198 or 64.1%) (P = 0.004).

A multivariable analysis was performed to consider cofactors that might have affected the efficacy assessments. Results were not affected by host sex, age, or skin Mf count at baseline. However, there was a significant intraclass correlation (ICC) effect for worm viability and fertility when multiple worms were assessed from individual study participants. ICC values for this outcome were 0.213 for worms overall, 0.079 after IA, 0.246 after IDA1, and 0.286 after IDA3. Results in Table 5 show that the unadjusted superior killing or sterilization effect of IDA1 relative to IA (the primary endpoint of the study) was not statistically significant after ICC was considered, although the trend remained. The enhanced killing/sterilizing effects of IDA3 vs. IA and of combined results from both IDA treatment groups vs. IA (secondary endpoints, see odds ratios in Table 5) were statistically significant even after ICC was considered. The difference in this outcome after treatment with IDA1 vs. IDA3 was not significant.

**Table 5:** Analysis of the effects of treatment on adult female *O*. *volvulus* worms adjusted for intraclass correlations for viability and fertility for multiple worms assessed within individual study participants.

Outcome	Treatment comparison	Odds ratio (95% CI)	Adjusted P-value
Fertile female *O*. *volvulus*	IA vs IDA1	1.66 (0.85–3.21)	0.134
	IA vs IDA3	2.23 (1.12, 4.44)	0.023
	IA vs both IDA groups	1.91 (1.09, 3.34)	0.023
	IDA1 vs IDA3	1.32 (0.64, 2.85)	0.430
Living female *O*. *volvulus*	IA vs IDA1	1.41 (0.84, 2.38)	0.192
	IA vs IDA3	1.45 (0.92, 2.29)	0.107
	IA vs Both IDA groups	1.44 (0.97, 2.15)	0.068
	IDA1 vs IDA3	1.03 (0.59, 1.79)	0.918

*Mixed-effects logistic regression models were conducted in SAS using PROC GLIMMIX. Participant level random effects were included in the model to adjust for multiple worms/person.

## Discussion

The onchocerciasis elimination program focuses on periodic mass distribution of IVM. While this strategy has been effective for preventing blindness and controlling dermatitis, annual IVM has little or no ability to kill or permanently sterilize adult worms. Clinical trials that have assessed the potential macrofilaricidal effects of more frequent treatment with ivermectin have yielded mixed results (for example, [24,29]). Clearly, a better macrofilaricidal treatment could accelerate the elimination timeline. We chose to study IDA because of its dramatically superior efficacy for clearing Mf from the blood of persons with LF. The comparator treatment (IA) is the regimen used by MDA programs for elimination of LF and onchocerciasis in countries that are coendemic for these diseases. Prior studies have shown that IA is no better for killing or permanently sterilizing *O*. *volvulus* adult worms than IVM alone [24,30]. Therefore, we designed this study to evaluate the tolerability and efficacy of IDA vs. IA for treatment of onchocerciasis after pretreatment with IVM alone.

There were safety concerns regarding the use of IDA in this study, because DEC was linked to serious ocular AEs when it was used to treat onchocerciasis in the past. However, those AEs were mainly seen in persons with heavy infections and after many doses of DEC [8]. We hypothesized that IDA would be safe if it were given after Mf counts in skin and eyes were reduced by IVM pretreatment, and our results suggest that this strategy was successful in this study. Although adverse events were recorded in about 30% of study participants, there were no significant differences in the frequency, severity, or type of AEs by treatment group, and no severe or serious TEAE was observed. Indeed, AEs were much less frequent in this study than after IVM pretreatment of the same population [19]. The tolerability assessment included 7 days of inpatient observation with follow-up through 3 months after treatment. It also included detailed ophthalmological examinations; this was the first onchocerciasis clinical trial to include OCT examinations to assess the posterior segment of the eye. Additional studies will be needed to confirm the tolerability of IDA after IVM pretreatment observed in this study. That is because this first use of IDA was performed in a relatively small number of people with light to moderate infections, and because persons with significant pre-existing ocular disease were excluded from the study. Ideally, future studies should enroll participants in areas with higher endemicity and infection densities. Future studies may also be able to relax ocular exclusion criteria, based on the lack of significant ocular AEs observed in this study.

Nodulectomy data suggest that a single dose or three daily doses of IDA killed or permanently sterilized about 40% of adult female worms relative to the number of living and fertile worms that were observed after the IA comparator treatment. However, this difference was not statistically significant for the predefined primary endpoint comparison (IA vs. IDA) when the analysis considered intraclass correlations of adult worm status within individual study participants. Secondary endpoint comparisons (IA vs. IDA3 or IA vs. combined IDA treatment groups) also suggested that IDA had significant macrofilaricidal activity relative to IA. The finding that calcified dead female worms were more common after IDA treatment than after IA provides additional evidence that IDA is more macrofilaricidal than the IA comparator treatment. The fact that many female worms remained alive and fertile after IDA1 and IDA3 probably explains why these treatments were not superior to IA for achieving long-lasting clearance of Mf from the skin.

This study was powered for detecting a macrofilaricidal effect of 50% relative to the comparator treatment, and this assumed an ICC of 0.2 for worm fertility for multiple worms evaluated within individual study participants. Our ability to demonstrate a robust macrofilaricidal effect was hampered by the lower than expected health of adult female worms in the IA comparator group. Our power calculation assumed that 30% of females would be alive and fertile after IA treatment, but the observed percentage was only 22.7%, and the majority of living worms observed in nodules from all treatment groups were old. This suggests that transmission of *O*. *volvulus* in the study region has been low for some time, and that could have reduced our ability to detect a significant macrofilaricidal effect of IDA. It is possible that the partial macrofilaricidal effects of IDA seen in this study are somehow related to the age and suboptimal health of worms in this study area. Future studies should be performed in areas with higher endemicity where *O*. *volvulus* worm populations are likely to be healthier and younger.

IDA results from this study are novel, because this is the only short-course oral treatment studied to date that appears to have superior macrofilaricidal activity relative to the current MDA regimen of IA (which is not macrofilaricidal). This begs the question of whether this finding has any practical significance. Although a single treatment with IDA1 or IDA3 clearly did not kill or sterilize a majority of adult female worms in this study, any treatment with macrofilaricidal activity superior to IA or IVM alone might accelerate onchocerciasis elimination across Africa. Also, multiple doses of combination treatments separated in time may improve macrofilaricidal activity.

MDA with IDA after IVM pretreatment would be more difficult to deploy than the currently recommended regimens (IA or IVM alone), but the extra effort could be justified if future studies demonstrate greater macrofilaricidal efficacy than that observed in this study. It is also important to mention that while there is a need for more effective MDA regimens, combination treatments could be very helpful for use cases other than MDA. For example, they might be used in endgame or mop-up situations, either as selective treatment of infected individuals (“test and treat”) or for focal MDA in hot-spot areas where routine MDA with IVM has failed to eliminate transmission.

## Summary and conclusions

This study showed that IDA was well tolerated in persons with light to moderate onchocerciasis infections after IVM pretreatment. Our results also strongly suggest that IDA has partial macrofilaricidal activity vs. *O*. *volvulus*. However, additional studies will be needed to confirm results from this pilot study. Experiences and results from this study will be useful for designing additional studies of combination treatments for onchocerciasis.

## Supporting information

S1 Protocol Study protocol. (PDF)

S1 Fig CONSORT diagram for the Part 1 study (screening, ivermectin pretreatment and subsequent assessments prior to enrollment in the current study). (JPG)

S1 Table Ocular adverse events (AE) following treatment. (DOCX)

S2 Table Nodulectomy results and effects of treatment on embryogenesis in female *O*. *volvulus* worms. (DOCX)

## References

[1] WHO. Progress report on the elimination of human onchocerciasis, 2019–2020. Wkly Epidemiol Rec. 2020;95:545–56. Epub 2020/11/14. .29130679

[2] Herricks JR, Hotez PJ, Wanga V, Coffeng LE, Haagsma JA, Basanez MG, et al. The global burden of disease study 2013: What does it mean for the NTDs? PLoS Negl Trop Dis. 2017;11(8):e0005424. Epub 2017/08/05. doi: 10.1371/journal.pntd.0005424 ; PubMed Central PMCID: PMC5542388. 28771480 PMC5542388

[3] Sauerbrey M, Rakers LJ, Richards FO. Progress toward elimination of onchocerciasis in the Americas. Int Health. 2018;10(suppl_1):i71–i8. Epub 2018/02/23. doi: 10.1093/inthealth/ihx039 . 29471334

[4] WHO. Progress in eliminating onchocerciasis in the WHO Region of the Americas: doxycycline treatment as an end-game strategy. Wkly Epidemiol Rec. 2019;93(47):414–9. Epub 2019/09/14.

[5] Traore MO, Sarr MD, Badji A, Bissan Y, Diawara L, Doumbia K, et al. Proof-of-principle of onchocerciasis elimination with ivermectin treatment in endemic foci in Africa: final results of a study in Mali and Senegal. PLoS Negl Trop Dis. 2012;6(9):e1825. doi: 10.1371/journal.pntd.0001825 ; PubMed Central PMCID: PMC3441490. 23029586 PMC3441490

[6] Zarroug IM, Hashim K, ElMubark WA, Shumo ZA, Salih KA, ElNojomi NA, et al. The First Confirmed Elimination of an Onchocerciasis Focus in Africa: Abu Hamed, Sudan. Am J Trop Med Hyg. 2016;95(5):1037–40. Epub 2016/11/04. doi: 10.4269/ajtmh.16-0274 ; PubMed Central PMCID: PMC5094213. 27352878 PMC5094213

[7] Ehrens A, Hoerauf A, Hubner MP. Current perspective of new anti-Wolbachial and direct-acting macrofilaricidal drugs as treatment strategies for human filariasis. GMS Infect Dis. 2022;10:Doc02. Epub 2022/04/26. doi: 10.3205/id000079 ; PubMed Central PMCID: PMC9006451. 35463816 PMC9006451

[8] Fischer PU, King CL, Jacobson JA, Weil GJ. Potential Value of Triple Drug Therapy with Ivermectin, Diethylcarbamazine, and Albendazole (IDA) to Accelerate Elimination of Lymphatic Filariasis and Onchocerciasis in Africa. PLoS Negl Trop Dis. 2017;11(1):e0005163. Epub 2017/01/06. doi: 10.1371/journal.pntd.0005163 ; PubMed Central PMCID: PMC5215784. 28056015 PMC5215784

[9] Thomsen EK, Sanuku N, Baea M, Satofan S, Maki E, Lombore B, et al. Efficacy, Safety, and Pharmacokinetics of Coadministered Diethylcarbamazine, Albendazole, and Ivermectin for Treatment of Bancroftian Filariasis. Clin Infect Dis. 2016;62(3):334–41. doi: 10.1093/cid/civ882 . 26486704

[10] King CL, Suamani J, Sanuku N, Cheng YC, Satofan S, Mancuso B, et al. A Trial of a Triple-Drug Treatment for Lymphatic Filariasis. N Engl J Med. 2018;379(19):1801–10. Epub 2018/11/08. doi: 10.1056/NEJMoa1706854 ; PubMed Central PMCID: PMC6194477. 30403937 PMC6194477

[11] Bjerum CM, Ouattara AF, Aboulaye M, Kouadio O, Marius VK, Andersen BJ, et al. Efficacy and Safety of a Single Dose of Ivermectin, Diethylcarbamazine, and Albendazole for Treatment of Lymphatic Filariasis in Cote d’Ivoire: An Open-label Randomized Controlled Trial. Clin Infect Dis. 2020;71(7):e68–e75. Epub 2019/10/24. doi: 10.1093/cid/ciz1050 ; PubMed Central PMCID: PMC7583415. 31641754 PMC7583415

[12] Dubray CL, Sircar AD, Beau de Rochars VM, Bogus J, Direny AN, Ernest JR, et al. Safety and efficacy of co-administered diethylcarbamazine, albendazole and ivermectin during mass drug administration for lymphatic filariasis in Haiti: Results from a two-armed, open-label, cluster-randomized, community study. PLoS Negl Trop Dis. 2020;14(6):e0008298. Epub 2020/06/09. doi: 10.1371/journal.pntd.0008298 ; PubMed Central PMCID: PMC7302858. 32511226 PMC7302858

[13] Jambulingam P, Kuttiatt VS, Krishnamoorthy K, Subramanian S, Srividya A, Raju HKK, et al. An open label, block randomized, community study of the safety and efficacy of co-administered ivermectin, diethylcarbamazine plus albendazole vs. diethylcarbamazine plus albendazole for lymphatic filariasis in India. PLoS Negl Trop Dis. 2021;15(2):e0009069. Epub 2021/02/17. doi: 10.1371/journal.pntd.0009069 ; PubMed Central PMCID: PMC7909694. 33591979 PMC7909694

[14] Supali T, Djuardi Y, Christian M, Iskandar E, Alfian R, Maylasari R, et al. An open label, randomized clinical trial to compare the tolerability and efficacy of ivermectin plus diethylcarbamazine and albendazole vs. diethylcarbamazine plus albendazole for treatment of brugian filariasis in Indonesia. PLoS Negl Trop Dis. 2021;15(3):e0009294. Epub 2021/03/30. doi: 10.1371/journal.pntd.0009294 ; PubMed Central PMCID: PMC8031952. 33780481 PMC8031952

[15] King CL, Weil GJ, Kazura JW. Single-Dose Triple-Drug Therapy for Wuchereria bancrofti—5-Year Follow-up. N Engl J Med. 2020;382(20):1956–7. Epub 2020/05/14. doi: 10.1056/NEJMc1914262 ; PubMed Central PMCID: PMC7175637. 32402169 PMC7175637

[16] WHO. Guideline: alternative mass drug administration regimens to eliminate lymphatic filariasis. King JD, editor: WHO/Department of control of neglected tropical diseases; 2017. 29565523

[17] Dadzie KY, Bird AC, Awadzi K, Schulz-Key H, Gilles HM, Aziz MA. Ocular findings in a double-blind study of ivermectin versus diethylcarbamazine versus placebo in the treatment of onchocerciasis. Br J Ophthalmol. 1987;71(2):78–85. doi: 10.1136/bjo.71.2.78 ; PubMed Central PMCID: PMC1041095. 3548811 PMC1041095

[18] Opoku NO, Bakajika DK, Kanza EM, Howard H, Mambandu GL, Nyathirombo A, et al. Single dose moxidectin versus ivermectin for Onchocerca volvulus infection in Ghana, Liberia, and the Democratic Republic of the Congo: a randomised, controlled, double-blind phase 3 trial. Lancet. 2018;392(10154):1207–16. doi: 10.1016/S0140-6736(17)32844-1 ; PubMed Central PMCID: PMC6172290. 29361335 PMC6172290

[19] Opoku NO, Gyasi ME, Doe F, Lew D, Hong AR, Chithenga S, et al. A Reevaluation of the Tolerability and Effects of Single-Dose Ivermectin Treatment on Onchocerca volvulus Microfilariae in the Skin and Eyes in Eastern Ghana. Am J Trop Med Hyg. 2021;106(2):740–5. Epub 2021/11/30. doi: 10.4269/ajtmh.21-0859 ; PubMed Central PMCID: PMC8832884. 34844204 PMC8832884

[20] CCRPS. Code of Federal Regulations and ICH Guidelines GCP Reference Guide 2022 [cited 2022 January 5th]. Available from: https://app.ccrps.org/courses/ich-gcp.

[21] MedDRA. Medical Dictionary for Regulatory Activities 2020 [cited 2020 04.20.]. Available from: https://www.meddra.org/.

[22] Weil GJ, Bogus J, Christian M, Dubray C, Djuardi Y, Fischer PU, et al. The safety of double- and triple-drug community mass drug administration for lymphatic filariasis: A multicenter, open-label, cluster-randomized study. PLoS Med. 2019;16(6):e1002839. doi: 10.1371/journal.pmed.1002839 . 31233507 PMC6590784

[23] Albiez EJ, Buttner DW, Duke BO. Diagnosis and extirpation of nodules in human onchocerciasis. Trop Med Parasitol. 1988;39 Suppl 4:331–46. Epub 1988/12/01. . 3067324

[24] Batsa Debrah L, Klarmann-Schulz U, Osei-Mensah J, Dubben B, Fischer K, Mubarik Y, et al. Comparison of repeated doses of ivermectin versus ivermectin plus albendazole for treatment of onchocerciasis—a randomized open-label clinical trial. Clin Infect Dis. 2019. Epub 2019/09/20. doi: 10.1093/cid/ciz889 . 31536624 PMC7428389

[25] Jolodar A, Fischer P, Buttner DW, Miller DJ, Schmetz C, Brattig NW. Onchocerca volvulus: expression and immunolocalization of a nematode cathepsin D-like lysosomal aspartic protease. Exp Parasitol. 2004;107(3–4):145–56. Epub 2004/09/15. doi: 10.1016/j.exppara.2004.06.006 . 15363940

[26] Fischer K, Dubben B, Debrah LB, Kuehlwein JM, Ricchiuto A, Debrah AY, et al. Histopathological evaluation of Onchocerca volvulus nodules by microscopy and by digital image analysis for the study of macrofilaricidal drug efficacy. Front Med (Lausanne). 2023;10:1099926. Epub 2023/02/24. doi: 10.3389/fmed.2023.1099926 ; PubMed Central PMCID: PMC9932808. 36817770 PMC9932808

[27] Buttner DW, Albiez EJ, von Essen J, Erichsen J. Histological examination of adult Onchocerca volvulus and comparison with the collagenase technique. Trop Med Parasitol. 1988;39 Suppl 4:390–417. Epub 1988/12/01. . 2852393

[28] Specht S, Brattig N, Buttner M, Buttner DW. Criteria for the differentiation between young and old Onchocerca volvulus filariae. Parasitol Res. 2009;105(6):1531–8. Epub 2009/09/29. doi: 10.1007/s00436-009-1588-5 ; PubMed Central PMCID: PMC2764059. 19784672 PMC2764059

[29] Gardon J, Boussinesq M, Kamgno J, Gardon-Wendel N, Demanga N, Duke BO. Effects of standard and high doses of ivermectin on adult worms of *Onchocerca volvulus*: a randomised controlled trial. Lancet. 2002;360(9328):203–10. 12133654 10.1016/S0140-6736(02)09456-4

[30] Awadzi K, Addy ET, Opoku NO, Plenge-Bonig A, Buttner DW. The chemotherapy of onchocerciasis XX: ivermectin in combination with albendazole. Trop Med Parasitol. 1995;46(4):213–20. Epub 1995/12/01. . 8826100

