# Supplementary material for: A randomized, open-label study of the tolerability and efficacy of one or three daily doses of ivermectin plus diethylcarbamazine and albendazole (IDA) versus one dose of ivermectin plus albendazole (IA) for treatment of onchocerciasis
Source: PLoS Negl Trop Dis. 2023 May 19;17(5):e0011365. doi: 10.1371/journal.pntd.0011365 (PMC10234528; doi:10.1371/journal.pntd.0011365)
Supplement: S1 Protocol — (PDF) [file pntd.0011365.s001.pdf]

Safety and efficacy of combination therapy with ivermectin, diethylcarbamazine, and albendazole (IDA) for individuals with onchocerciasis

Protocol Identifier: DOLF_IDA Oncho Safety_Ghana

Type: Open label randomized clinical trial

Principal Investigator:

Nicholas Opoku, MB, CHB, MSC [1]

Co-Principal Investigators:

Christopher L. King, MD, PhD [2]

Gary J. Weil, MD [3]

Co-Investigators:

Peter U. Fischer, PhD[3]

Catherine Bjerum, MD, MPH[2]

Felix Doe, MD, MPH[4]

Michael E. Gyasi, MD, MSC [5]

Augustine R. Hong, MD [3]

Eric Kanza, MD[6]

Philip J. Budge MD, PhD [3]

[1] School of Public Health, University of Health and Allied Sciences, Hohoe, Ghana
[2] Center for Global Health & Diseases, Case Western Reserve University, USA
[3] Washington University School of Medicine, St Louis, USA
[4] Municipal Health Directorate, Hohoe, Ghana
[5] St. Thomas Eye Hospital, Accra, Ghana
[6] Université Catholique du Graben

Initial Protocol: v3.0, 28 JUNE 2019
Current Protocol: v3.1, 21 MAY 2020

Summary of Changes from Previous Version:

Affected Section (s)	Summary of Revisions Made	Rationale
5.5	Section was added to describe modified procedures and potential risks to participants in conducting trial during COVID-19 pandemic	These additions to the protocol describe in detail the modifications being made to protect participant safety during the COVID-19 pandemic. No changes are being made to the actual study design of the trial.

Version: **3.1 Statement of Compliance**

Safety and efficacy of combination therapy with ivermectin, diethylcarbamazine, and albendazole (IDA) for individuals with onchocerciasis

DOLF_IDA Oncho Safety_Ghana v[3.1] [20/MAY/2020]

PACTR Registration Number: PACTR201906665550709

This study will be carried out in accordance with Good Clinical Practice (GCP) as required by the:

- U.S. Code of Federal Regulations applicable to clinical studies (45 CFR 46) https://www.hhs.gov/ohrp/regulations-and-policy/regulations/45-cfr-46
- ICH GCP E6 Completion of Human Subjects Protection Training https://grants.nih.gov/grants/guide/notice-files/NOT-OD-16-148.html
- Guidelines for Good Clinical Practice in Ghana https://fdaghana.gov.gh/index.php/clinical-trials-department/
- Bill and Melinda Gates Foundation "grant agreement" terms and conditions

SIGNATURES
I have read the protocol, including the appendices, and I agree that it contains all necessary details for me and my staff to conduct this study as described. I agree that this study will be conducted according to all stipulations of the protocol, including all statements regarding confidentiality and according to local legal and regulatory requirements and to the principles outlined in applicable U.S. federal regulations, Ghanaian and ICH guidelines.

I will provide all study personnel participating in the study under my supervision copies of the protocol and access to all study related information provided by the DOLF project. I will discuss with them to ensure they are fully informed about the study procedures.

Principal Investigator: ______________________________

Name/Title (Print/Type)

Signed: ______________________________ **Date:** ______________

NOTE: The PI and co-PIs should have signed investigator agreements on file.

Table of Contents

Key Study Contacts **7**

List of Abbreviations **9**

1 PROTOCOL SUMMARY **11**

2 BACKGROUND INFORMATION AND SCIENTIFIC RATIONALE **12**

2.1 Background **12**

Ocular disease associated onchocerciasis and exacerbation with treatment 12

2.2 Literature Review **13**

Prior treatments for onchocerciasis using diethylcarbamazine and ivermectin 14

2.3 Problem Statement **15**

Better treatment is needed to eliminate Onchocerciasis 15

Triple drug treatment for elimination of Lymphatic Filariasis in Africa 16

Gaps in knowledge about onchocercal eye disease 16

New technology to better understand onchocercal ocular disease 16

3 HYPOTHESES **17**

4 STUDY OBJECTIVES AND PURPOSE **17**

4.1 Study Design **19**

4.2 Inclusion and Exclusion Criteria **19**

Inclusion Criteria 19

Exclusion Criteria 20

4.3 Participant Exclusion from Routine MDA **21**

4.4 Randomization **21**

4.5 Treatment masking **22**

4.6 Study Procedures **22**

Parasitological Evaluations 23

Nodulectomies 23

Wound treatment for Nodulectomy 24

Ocular Examinations: 24

General health examination and skin evaluation. 25

4.7 Participant Treatment **25**

4.7.1 Plan for Re-dosing 26

4.7.2 Concomitant Medications 26

4.8 Assessment of Adult Filarial Worms **26**

4.9 Study Duration and Timeline **27**

4.10 Investigational Product Supply and Storage **28**

5 POTENTIAL RISKS AND BENEFITS **28**

5.1 Risks of Skin Snips: **28**

5.2 Risk of ocular exams **28**

5.3 Risk of study medications: **29**

5.4 Risks of nodulectomy **30**

5.5 Conducting the trial during the Covid-19 pandemic **30**

5.6 Study Benefits **31**

Benefits to Individuals 31

Public health benefit 31

5.7 Study Compensation **32**

Compensation for Loss of Earnings 32

Compensation for Research Related Injuries......32

6 INSTRUCTIONS FOR SPECIMEN PREPARATION, HANDLING, AND STORAGE...... 32
6.1 Specimen and Sample Handling...... 32
6.2 Specimen Shipment...... 32

7 SAFETY REPORTING AND SAFETY MONITORING...... 33
7.1 Definitions...... 33
Adverse Events (AEs)......33
Ocular Adverse Events (OAEs)......33
Serious Adverse Event (SAE)......34
Ocular SAEs......34
Unexpected......34
Expedited Safety Report......34
7.2 Assessment of Adverse Events...... 34
7.3 Serious Adverse Event (SAE) Assessment and Management...... 35
7.4 Reporting of Pregnancy...... 35
7.5 Data Safety Monitoring Board...... 36
7.6 Interim Analyses...... 36
7.7 Procedures for Pausing the Trial...... 36

8 STATISTICAL CONSIDERATIONS...... 37
8.1 Sample Size Determination (Power calculation)...... 37
8.2 Data Analysis Plan...... 37
Efficacy analysis for primary outcome......37
Efficacy analysis for secondary outcomes......38
Safety......38
8.3 Interim and Final Reports...... 38

9 DATA HANDLING...... 39
9.1 Types of Data Collected...... 39
Enrollment Data......39
Laboratory Results......39
Participant Monitoring and Follow-up......40
Adverse Event Evaluation......40
9.2 Study Records Retention...... 40
9.3 Participant Key/Participant List...... 40
9.4 Source Documents...... 40

10 FUTURE USE OF STORED SPECIMENS...... 41

11 ETHICAL ISSUES...... 41
11.1 Informed Consent Process...... 41
11.2 Comprehension of Informed Consent...... 41
11.3 Provisions for Subjects from Vulnerable Populations...... 42
Plan to avoid treating pregnant women......42
Plan for Inclusion of Illiterate Subjects......42
Plan for Inclusion of Non-English Speaking Individuals......42
Minors......43
11.4 Alternatives to participation...... 43
11.5 Withdrawal...... 43
11.6 Participant Privacy...... 43
11.7 Plan for Subjects at the End of the Protocol...... 44

12 RESPONSIBILITIES 44
12.1 Good Clinical Practice 44
12.2 Clinical Trial Monitoring 44
12.3 Institutional Review Board (IRB)/Ethics Review Committee (ERC) 44
12.4 Data Ownership 45
12.5 Financial Disclosure 45
12.6 DOLF Team Technical Support 45
13 LITERATURE REFERENCES 45
14 APPENDICES 49

Key Study Contacts

Principal Investigator:

Nicholas Opoku, MB, CHB, MSC
University of Health and Allied Sciences
Hohoe, Ghana
+233 244776668
noopoku@uhas.edu.gh

Co-Principal Investigators:

Christopher L. King, MD, PhD
Case Western Reserve University
Center for Global Health and Diseases
10900 Euclid Ave LC: 4983
Cleveland, OH 44106
+1 216-368-4817
cxk21@case.edu

Gary J. Weil, MD
Washington University School of Medicine
4444 Forest Park, Ste. 2212
St. Louis, MO 63110
+1 314-454-7787
gary.j.weil@wustl.edu

Co-Investigators:

Peter U. Fischer, PhD
Washington University School of Medicine
4444 Forest Park, Ste. 2212
St. Louis, MO 63110
+1 314-454-7876
pufischer@wustl.edu

Catherine Bjerum, MD, MPH
Case Western Reserve University
Center for Global Health and Diseases
10900 Euclid Ave LC: 4983
Cleveland, OH 44106
+1 602-828-1669
cmbjerum@msn.com

Felix Doe, MD, MPH
Municipal Health Directorate
PO Box 27
Hohoe, Ghana
+233 2445118342
felixdoe@yahoo.co.uk

Michael Gyasi, MD, MSC
St. Thomas Eye Hospital,
Accra, Ghana
+233 246866580
mikegyasi67@gmail.com

Augustine R. Hong, MD
660 South Euclid Avenue
Campus Box 8096
Saint Louis, MO 63110
+1 314-362-3937
ahong22@wustl.edu

Eric Kanza, MD
Centre de Recherche Clinique de Butembo (CRCB)
Université Catholique du Graben (UCG)
Butembo, Democratic Republic of the Congo
+243 998 30 93 53 / +243 81 21 12 695
kanzaeric@gmail.com

Philip J. Budge MD, PhD
Washington University School of Medicine
4444 Forest Park, Ste. 2212
St. Louis, MO 63110
+1 314-747-5532
pbudge@dom.wustl.edu

Pharmacists:

Lipe Johnson Gavu
Hohoe Municipal Hospital
Hohoe, Ghana
+233 (20) 1230177 LipeGavu@GMail.com.

Local Medical Monitor

Emmanuel Senyo Kasu, MB ChB, MPH,
Head, Public Health Dept.
Volta Regional Hospital, Ho, Ghana
+233 244087676
eskasu@uhas.edu.gh

Local Study Monitor

Margaret Williams
Margan Clinical Research Organization
67 Eastern Bypass
Aburi, Ghana
+233 249 333 809
mwilliams@margancro.org;
mywilliams015@gmail.com

Study Sponsor:

Washington University School of Medicine
Jennifer Klenke (Contact)

4444 Forest Park, Ste. 2212
St. Louis, MO 63110
+1 (314) 286-2112
jklenke@wustl.edu

Laboratory:
Weil Lab
Washington University in St. Louis
4444 Forest Park Blvd
St. Louis MO 63110
+1(314) 454-7783

List of Abbreviations

**AE**	Adverse Event
**ALB**	Albendazole
**CFR**	Code of Federal Regulations
**CTCAE**	Common Terminology Criteria for Adverse Events
**DEC**	Diethylcarbamazine
**DOLF**	Death to Onchocerciasis and Lymphatic Filariasis
**DSMB**	Data Safety Monitoring Board
**EDC**	Electronic Data Capture System
**ERC**	Ethics Review Committee
**FDA**	Food and Drug Administration
**GCP**	Good Clinical Practice
**GHS**	Ghana Health Service
**IATA**	International Air Transport Association
**ICH**	International Conference on Harmonisation
**IDA**	Ivermectin, Diethylcarbamazine, and Albendazole
**I/IDA**	IDA following ivermectin pre-treatment
**IRB**	Institutional Review Board
**IVM**	Ivermectin
**LF**	Lymphatic Filariasis
**MDA**	Mass Drug Administration
**Mf**	Microfilaria(e)
**OCRC**	Onchocerciasis Chemotherapy Research Centre
**OCT**	Optical Coherence Tomography
**PI**	Principal Investigator
**POC**	Point of Care
**RPE**	Retinal Pigment Epithelium

**SAE**	Serious Adverse Event
**UHAS**	University of Health and Allied Sciences
**UNID**	Unique Study Identification Number
**VF**	Visual Field
**WHO**	World Health Organization

1 PROTOCOL SUMMARY

**Study Title:**	Safety and efficacy of triple drug therapy for individuals infected with *Onchocerca volvulus*
**Type of Study:**	Open label, randomized clinical trial
**Study Drugs:**	Diethylcarbamazine citrate (DEC) tablets - $C_{16}H_{29}N_3O_8$<br>Ivermectin tablets - $C_{48}H_{74}O_{14}$<br>Albendazole tablets - $C_{12}H_{15}N_3O_2S$
**Population:**	Participants from the study '*Microfilarial Clearance from the Eye and Ocular Changes Associated with Ivermectin Treatment in Individuals with Onchocerciasis'* will be invited to participate in the study described in this protocol. These participants were recruited from the Nkwanta North District in the Volta region of Ghana located at the northeastern part of Ghana. The population of Nkwanta North District, according to the 2010 Population and Housing Census, is 64,553.
**Number of Participants:**	Up to 300 eligible participants
**Study Duration**	24 months
**Duration of Participant Participation**	Participants will be followed for 18 months after their first treatment.
**Primary Objectives:**	**Safety:** To compare rates and types of severe adverse events (grade 3 or higher) that occur within 7 days following 1 day or 3 days of triple drug treatment (DEC with ivermectin and albendazole; "IDA") with the comparator regimen of 1 day of treatment with ivermectin and albendazole in persons with onchocerciasis at least six months after pretreatment with ivermectin alone (in a preceding study).<br>**Efficacy:** To compare the effect of three treatment regimens for killing or sterilizing adult female *O. volvulus* worms based on the percentage of all adult female worms in nodules that are alive with embryos in the uterus 18 months after treatment.

Secondary Objectives:	**Safety:**<br>1. To compare rates of adverse events grade 3 or higher by treatment group that occur within 7 days of treatment in the subset of participants who have intraocular microfilariae just prior to treatment with IDA<br>2. To compare rates of ocular adverse events (any grade) by treatment group that occur within 3 months of treatment with IDA<br>**Efficacy**<br>1. To compare the effect of three treatment regimens for killing adult female *O. volvulus* worms based on the percentage of all adult female worms in nodules that are alive 18 months after treatment with IDA<br>2. To compare the effects of three treatment regimens for complete clearance of microfilariae from the skin. 3, 12 and 18 months after treatment with IDA<br>3. To compare the effects of three treatment regimens for preventing reappearance of microfilariae in the skin at 12, and 18 months after treatment with IDA.

2 BACKGROUND INFORMATION AND SCIENTIFIC RATIONALE

2.1 Background

Ocular disease associated onchocerciasis and exacerbation with treatment

Onchocerciasis is caused by the filarial nematode *Onchocerca volvulus* and transmitted by the bite of *Simulium* blackflies that breed in rivers. Larval parasites (Mf) migrate through the skin and can cause severe skin and ocular diseases ("river blindness"). The World Health Organization (WHO) estimates that at least 25 million people are infected worldwide, affecting mainly 31 countries in sub-Saharan Africa, including Ghana [1, 2]. Onchocerciasis is the world's second leading infectious cause of blindness. An estimated one million people have been blinded or have severe visual impairment due to onchocerciasis [1, 2]. Mass drug administration (MDA) with ivermectin for onchocerciasis has significantly reduced the burden of infection and associated ocular disease to the point where ocular disease is now uncommon in most endemic areas [2, 3]. Improved treatments that kill or sterilize adult *O. volvulus* worms would accelerate elimination of the infection.

2.2 Literature Review

Ocular pathology related to onchocerciasis was previously more common in savannah areas of West Africa and in Central America, and this has been attributed to various factors, such as localization of nodules in the upper part of the body, vector species, Mf burden, and parasite strains [4, 5]. Onchocerciasis is a parasitic worm infection transmitted by *Simulium* black flies. The flies breed near rivers/streams, and biting rates are highest near the breeding sites. The infection is common in forested and savannah areas in West Africa that are drained by fast flowing rivers and streams. Ocular pathology (including blindness) was previously more common in savannah areas. Reasons for this are not fully understood, but we provided two references in the text to demonstrate what is known [4, 5]**.**

The actual route of entry of Mf into the eyes is not known, but proposed routes include the sheaths of the posterior ciliary arteries and nerves, the blood stream, the bulbar conjunctiva with entry through the limbus, and along the orbital septum and the cheek ligaments [6, 7, 8]. The most common ocular lesion involves the cornea, that begins with "fluffy" or "snow-flake" stromal opacities (i.e., punctate keratitis), which later coalesce with fibrosis (i.e., sclerosing keratitis). The anterior chamber can be invaded by Mf, and death of Mf can be associated with uveitis with formation of synechiae, cataract and glaucoma. The lesions in the posterior segment of the eye include atrophy of the retinal pigment epithelium (RPE), choroidoretinal scarring, subretinal fibrosis and optical neuritis and post-neuritic optical atrophy which can be associated with permanent visual loss [9, 10]. The main pathological changes seen in the posterior chamber of the eye are related to local invasion and host reactions to death of Mf within the retinal tissue. Some researchers have hypothesized that cross-reactive antibodies induced by *O. volvulus* antigens (Ov39) and the RPE antigen (hr44) may contribute to posterior segment diseases [11, 12, 13, 14, 15, 16]. Indeed there is evidence of persistent ocular deterioration in subjects even following clearance of Mf from the anterior chamber of the eye [17], or after years of repeated IVM treatment [18]. Whether these late ocular changes are a) the result of autoimmune responses, b) persistence of Mf or their products in the posterior chamber, or c) a parasite-independent disease progression is unknown. This is partly due to limitations in technology available to assess the retina when these studies were performed. New methods are now available to better assess the posterior chamber of the eye. Such techniques have not been previously used to study ocular pathology in subjects with onchocerciasis (see below).

Onchocercal ocular disease has decreased dramatically with widespread use of IVM. Because IVM alone has no permanent effect on the adult worms and adult worms can live up to 15 years, the use of IVM alone for onchocerciasis elimination has only been successful in areas with low transmission, and/or in geographically isolated areas such as in the Americas [19], Senegal and Mali [20] or Northern Sudan [21]. However, there also examples where 15 or more rounds of IVM were not sufficient to interrupt transmission [22, 23, 24]. There is an urgent need to find new drugs or novel combinations of existing drugs that can partially or completely sterilize/kill adult worms for elimination of onchocerciasis in Africa to occur [25, 26]. Studies of new treatments should use the best available methods to assess potential ocular adverse events following treatment.

The prevalence of Mf in the eye is correlated with microfiladermia levels and overall community prevalence of onchocerciasis [6, 7, 8, 27]. Intraocular Mf are uncommon in persons who live in areas with microfiladermia rates below 20%, and they are uncommon in persons with <10 Mf/mg skin. Additionally, high intraocular Mf counts are not always associated with high rates of eye disease. For example, 44% of examined individuals in a hyper-endemic focus in Western Uganda had Mf in the anterior chamber, but sclerosing keratitis and vision loss were uncommon[5].

Prior treatments for onchocerciasis using diethylcarbamazine and ivermectin

The use of DEC and its associated adverse events in individuals with heavy infections with oncherciasis was recently reviewed by the study team[26]. The discussion below summarizes some key points from that publication. Prior to 1987, an 8 to 14 day course of diethylcarbamazine (DEC) was commonly used to treat onchocerciasis. DEC was an effective microfilaricide, but it occasionally was associated with the development of severe inflammatory responses—both systemic and dermal—caused by the death of Mf. This is referred to as a Mazzotti reaction. DEC also caused adverse events in the eye, with punctate and sclerosing keratitis, limbitis, anterior uveitis, and chorioretinitis. It sometimes caused optic neuritis with edema and visual impairment in individuals with heavy *O. volvulus* infection who received high doses of the drug for prolonged periods [9, 28].

During the 1980's a single dose of IVM was shown to be more effective than DEC for clearing the Mf from the skin with minimal ocular adverse effects [29], and it has been the treatment of choice since that time. IVM reduces *O. volvulus* Mf numbers in skin within a few days with a nadir of skin Mf between 1 and 3 months post-treatment. Mf may reappear in the skin as early as 3 months post-treatment [30]. Mf clearance from the eye following IVM treatment is slower by several months than clearance observed in the skin [6, 31]. The nadir of Mf clearance from the anterior chamber of the eye starts 6 months post-treatment based on unpublished results from heavily infected subjects in the Volta region of Ghana with ocular Mf (Table 1), and that reduction persisted out to 18 months after treatment [32].

Table 1. Kinetics of Mf clearance from the anterior chamber of the eye following IVM treatment

Visit (Days/months post IVM 150 mcg/kg treatment)	*Subjects with ≥ 1 Mf in the anterior chamber of both eyes before and following a single dose of IVM n/N (%)*
Day 0 (pretreatment)	205/205 (100)
Day 3	145/174 (83.3)
Day 4	21/23 (91.3)
Month 1	90/201 (44.8)
Month 6	14/200 (7.0)
Month 12	16/195 (8.2)
Month 18	13/160 (8.1)

The clearance of Mf from the posterior chamber of the eye is poorly understood because it is more difficult to visualize this part of the eye. Several studies have shown that anterior segment lesions such as punctuate keratitis, sclerosing keratitis, iridocyclitis improved after IVM [33, 34], but there are conflicting data about the effectiveness of IVM for improving vision or preventing visual loss related to posterior segment lesions.

The Fischer et al. review also discussed the possibility for re-introducing DEC in combination with ivermectin and albendazole, referred to as IDA for treatment of onchocerciasis [26]. The reason for this is that IDA has been found to be much more efficacious for treatment of lymphatic filariasis (LF) [35]. This study along with a large multicenter safety study (manuscript development in progress) resulted in the World Health Organization changing its guidelines to recommend IDA for LF outside Africa [36]. Based on this recommendation Merck Sharp & Dohme Inc agreed to increase

their donation of ivermectin by 100 million doses per year for use of IDA in MDA programs for LF outside of sub-Saharan Africa [37]. LF is often co-endemic with onchocerciasis in sub-Saharan Africa. This raises the important question of whether IDA can be safely used for LF elimination in coendemic areas. We hypothesize that IDA will be safe for use in persons with onchocericasis if dermal and ocular Mf are cleared or greatly reduced by pretreatment with ivermectin alone

Many studies have examined Mf clearance from the skin after IVM treatment and these have been summarized in a meta-analysis [38]. These results show microfilaridermia are reduced by half after 24 h, by 85% after 72 h, by 94% after 1 week, and by 98-99% after 1-2 months . Mf reappear in the skin as early as 3 months post-treatment [38].

Ocular Mf in the anterior chamber of the eye can be readily observed with a slit lamp. Using slit lamp examination Mf clearance from the eye following ivermectin treatment lags that observed in the skin by several months, but most treated subjects completely clear Mf from their eyes by three or six months after treatment [6, 33, 34]. This was highlighted in a recent study where individuals with >10 Mf in the anterior chamber and treated with a single dose of 150 ug/kg of ivermectin and examined at 4 day, 1, 6, 12 and 18 months post-treatment show that Mf reached a nadir in mean Mf numbers in the anterior chamber at 6 months with little increase at 12 and 18 months [39]. Mean percent reduction in Mf in the anterior chamber was 97±12%, N=74 at 12 months [39]. Among the few individuals that failed to clear ocular Mf in anterior chamber, almost all ocular Mf were immobile and probably dead.

A few older studies describe lesions associated with Mf in the posterior segment of the eye [9, 33, 40, 41], which are though to cause most of the irreversible causes of visual loss. These changes primarily involved optic nerve disease and choroido-retinal changes, particularly retinal atrophy. However, Mf were rarely observed in the posterior segment of eye, even among individuals with high levels of microfiladermia. Several studies have shown that IVM may improve anterior segment lesions such as punctuate keratitis, sclerosing keratitis, iridocyclitis [33], but there are conflicting data about the effectiveness in preventing visual loss from posterior segment lesions [18]. The current study will examine whether IDA, which includes DEC, can be safely used following a pretreatment dose of IVM to reduce Mf in the eye.

2.3 Problem Statement

Better treatment is needed to eliminate Onchocerciasis

Programs for control of onchocerciasis through community directed treatment with IVM as a form of MDA have been in place for almost 30 years. IVM is effective for clearing Mf and it temporarily sterilizes adult female worms, but it is not a microfilaricide and does not kill adult worms. For that reason, MDA with ivermectin must be repeated for the reproductive life of the adult worms, which is 10-15 years. Thus, there is a widely recognized need for drug(s) that can kill or permanently sterilize adult worms. A new, safe, short-course treatment that kills or sterilizes adult worms would be a breakthrough that could accelerate onchocerciasis elimination in Africa.

The enhanced activity of IDA against filarial worms that cause LF suggests that this combination should be tested against *O. volvulus*. However, a single dose of IDA may be insufficient to kill/sterilize adult *O. volvulus.* Therefore, it is important to initially test the impact of a short multi-dose regimen of IDA. Three days of treatment would approach the practical limit for an MDA regimen.

Triple drug treatment for elimination of Lymphatic Filariasis in Africa

Recent studies have shown that a single co-administered dose of IVM, DEC and ALB (IDA) is far superior for long-term reduction and elimination of blood Mf of human lymphatic filariasis (LF) compared to the standard treatment of a) IVM and ALB or b) DEC and ALB, indicating permanent sterilization of adult worms. This combination therapy should require only one or two annual rounds of MDA to interrupt transmission (with high compliance) in areas with moderate endemicity, while five or more rounds are required with prior 2-drug regimens. WHO has recently recommended use of IDA for LF elimination in regions that are not coendemic for onchocerciasis or loiasis and that are not currently on track to eliminate LF by 2020 (e.g. Madagascar, Zimbabwe). However, these countries represent only 10% of the population at risk for LF in the AFRO region. If IDA can be safely used in persons with onchocerciasis after pretreatment with IVM, it could be very useful for eliminating LF in countries that are coendemic for LF and onchocerciasis even if the combination is not effective for killing or sterilizing adult *O. volvulus* worms. While this study is not powered to demonstrate safety of IDA in persons with onchocerciasis, it will provide preliminary safety data that could lead to larger safety studies later.

The WHO does not currently recommend use of IDA in areas with co-endemic LF and onchocerciasis. That is because one of the medicines in the IDA combination (diethylcarbamazine, also known as DEC) can cause severe/serious ocular adverse events in persons with high intensity infections with Mf in the eyes. Ivermectin pre-treatment (required for inclusion in this study) will reduce or completely clear Mf from the eyes of participants before they are treated with IDA in this study. Thus, pre-treatment should make it safe for participants to be treated with IDA. That would lead to accelerated elimination of LF in Africa, and IDA may also be better than currently available treatments used for elimination of onchocerciasis**.**

Gaps in knowledge about onchocercal eye disease

Although *O. volvulus* Mf can be readily observed in the anterior segment of eye using a slit lamp, it is challenging to visualize them in the posterior segment by previously used methods such as fundus photography, fluorescein angiography or indirect ophthalmoscopy. It is very uncommon for Mf to be in the posterior segment if the anterior chamber is clear**.** It is widely accepted among onchocerciasis researchers that the risk of ocular adverse events following DEC treatment is believed to be related to the number of Mf in the eye prior to treatment. Ophthalmologists believe that significant ocular adverse events (AEs) are very unlikely to occur if few Mf are present in the anterior chamber (Todd Margolis, Department Chairman of Ophthalmology at Washington University in St. Louis; Hugh Taylor, onchocerciasis expert and Professor of Ophthalmology, University of Melbourne, Australia; Yankum Dadzie, a retired Ghanaian ophthalmologist and former Director of the WHO Onchocerciasis Control Programme).

New technology to better understand onchocercal ocular disease

The methods currently available to evaluate ocular disease in patients with onchocerciasis include slit lamp, direct and indirect ophthalmoscopy, fundus photography, and fluorescein angiography. The latter test requires intravenous injection of a dye that sometimes causes acute adverse events (1 – 9%) that can include nausea, vomiting, allergic reactions, and (rarely) hypertensive crisis or myocardial infarction. Slit lamp examination is useful for demonstrating the presence of Mf in the anterior segment of the eye, including the cornea and anterior chamber, and in the vitreous, and for evaluating the presence and severity of intraocular lesions. Optical coherence tomography (OCT) is

the current method of choice for detailed assessment of the posterior segment and retina for many ocular diseases. It is a rapid, simple, precise and non-invasive imaging method that uses low intensity laser light to capture three-dimensional images of both the anterior and posterior segments of the eye with micrometer-resolution [42]. OCT examinations are safe and they only require a few minutes. OCT has not been used previously in patients with onchocerciasis. It should allow examiners to visualize Mf in eye in the visual axis of the eye, particularly the posterior segment, and to evaluate retinal anatomy in a way that is superior to fluorescein angiography with no risk of adverse events.

OCT is not a new technology. It has been used to evaluate the posterior segment of the eye for more than 20 years, and several centers in Ghana have OCT machines. However, since most ophthalmological studies of onchocerciasis were performed prior to development of OCT, this will be the first study to use of OCT in patients with onchocerciasis. OCT is less invasive and provides a more detailed assessment of retinal anatomy than fluorescein angiography, which was used in past studies of posterior segment disease related to onchocerciasis. OCT is the state of the art, and it is appropriate that we use it in this study to carefully assess potential adverse events following treatment.

The slit lamp is better for assessing the anterior segment of the eye, and OCT is superior for the posterior segment. These are complementary tools, and both will be used in this study for every eye examination. Ocular exams will occur the day prior to treatment, on Day 3 after treatment (during the 7 day observation period), and on Day 7 of the observation period.

3 HYPOTHESES

1. Triple drug therapy with ivermectin, diethylcarbamazine and albendazole (IDA) (following ivermectin pretreatment) is more effective for killing or sterilizing adult female *O. volvulus* worms than ivermectin plus albendazole.
2. Triple drug therapy with ivermectin, diethylcarbamazine, and albendazole (following ivermectin pretreatment) is as safe as IVM/ALB alone in persons with onchocerciasis.

4 STUDY OBJECTIVES AND PURPOSE

This study will provide preliminary data on the safety of IDA treatment in persons with onchocerciasis when it is administered after pre-treatment with IVM to clear or greatly reduce microfilariae from the skin and eyes. Widespread use of IDA following IVM pretreatment (I/IDA) has the potential to greatly accelerate elimination of LF in African countries that are coendemic for LF and onchocerciasis.

Note that the sample sizes per treatment group in this study are not sufficient to conclusively demonstrate that IDA after IVM pretreatment is safe enough for widespread use in communities. Therefore, the safety study should be considered to be exploratory, and larger studies would be needed to formally establish safety of a pretreat/treat strategy with ivermectin followed by IDA ("I/IDA"). Having said that, a small exploratory safety study like this is an appropriate first step, because it should generate sufficient data to justify (or rule out) performance of a larger safety study later.

This study will also assess the efficacy of IDA for killing and sterilizing adult filarial worms. An improved macrofilaricidal treatment would be a major advance for the global program to eliminate onchocerciasis. Since the safety and efficacy objectives are both very important, we have included dual primary objectives for the study.

**Primary Objectives:**	**Safety:** To compare rates and types of severe adverse events (grade 3 or higher) that occur within 7 days following 1 day ***or*** 3 days of treatment with triple drug treatment ("IDA" = DEC with ivermectin and albendazole) with the comparator regimen of 1 day of treatment with ivermectin and albendazole (IA) in persons with active *Onchocerca volvulus* infections after pretreatment with ivermectin alone.<br><br>**Efficacy:** To compare the effect of three treatment regimens (1 day of IDA, 3 days of IDA, or IA) for killing or sterilizing adult female *O. volvulus* worms based on the percentage of all adult female worms in nodules that are alive with embryos in the uterus 18 months after treatment.
**Secondary Objectives:**	**Safety:**<br>1. To compare rates of adverse events grade 3 or higher by treatment group that occur within 7 days of treatment in the subset of participants who have intraocular microfilariae just prior to treatment with IDA to those who do not have intraocular microfilariae<br>*2.* To compare rates of ocular adverse events (any grade) by treatment group that occur within 3 months of treatment with IDA<br><br>**Efficacy**<br>1. To compare the effect of three treatment regimens for killing adult female *O. volvulus* worms based on the percentage of all adult female worms in nodules that are alive 18 months after treatment.<br>2. To compare the effects of three treatment regimens for complete clearance of microfilariae from skin snips 3, 12 and 18 months after treatment with IDA.<br>3. To compare the effects of three treatment regimens for preventing reappearance of microfilariae in the skin at 12, and 18 months after treatment with IDA.

4.1 Study Design

This is an open label, randomized clinical trial will be conducted in Ghana. The goal will be to enroll up to 300 eligible participants with microfiladermia. Some participants who have met the inclusion criteria (see Study Enrollment, below) will have intraocular Mf visible by slit lamp at baseline prior to IVM pre-treatment. Participants will be recruited from an on-going related study that is evaluating the kinetics of Mf clearance from the eye and skin after treatment with IVM. Participants will be treated with ivermectin at baseline in this study and then randomized into three treatment arms 6 months (with a window of 5.5 months to 12 months) after treatment with IVM:

1. IVM with albendazole (the comparator)
2. Asingle dose of IDA
3. Three daily doses of IDA

Each arm will be stratified with respect to the presence ocular Mf six months after their first IVM pre-treatment (i.e. in "Part I", a precedingstudy) or at the baseline assessment in the Part II study. Participants will be closely monitored to determine whether the treatments are safe. Efficacy will be assessed by performing skin snips for detection of Mf and by histological examination of adult worms in onchocercal nodules surgically removed 18 months after treatment with IDA.

All participants will be treated again with ivermectin at the time of consenting for the Part II study to further ensure that they have low skin and ocular Mf counts at the time of enrollment and randomization to treatment arms. Treatment with ivermectin is widely accepted as a standard of care for MDA programs, and should not present undo risk.

Study Enrollment

Participants will be recruited from an on-going related study of the kinetics of Mf clearance from skin and eyes after ivermectin treatment (Part I). Participants from that study with baseline skin Mf counts $\geq$ 3 Mf/mg will be invited to participate in this "Part II" study.

Study Population - Participants will be recruited from the Nkwanta North and South Districts in the Volta region of Ghana. Most participants will be recruited from Kpassa town or villages in Kpassa sub-district along the Kpassa river basin that are endemic for onchocerciasis. Residents are mainly of the Konkomba ethnic group. The local language is Konkomba, however most residents also speak and understand the Twi language**.** Bi-annual MDA with IVM in the area started in October 2017. The target number of participants for the study will be up to 300.

4.2 Inclusion and Exclusion Criteria

Inclusion Criteria

1. Men and women 16 – 70 years (at the time of enrollment; ±2 years) of age previously enrolled in the Part 1 study and residing in the area of the study.
2. Participants must have at least 1 palpable subcutaneous nodule (onchocercoma).
3. Participants with baseline skin Mf counts ≥ 3Mf/mg at the time of enrollment into the Part 1 study (Part I, see above).

Exclusion Criteria

1. Pregnant and breastfeeding mothers within 1 month of giving birth
2. Severe eye disease at baseline including uveitis, severe glaucoma, , severe keratitis, and/or cataracts that interfere with visualization of the posterior segment of the eye as well as the list of ocular diseases as outlined below Individuals who are excluded with significant ocular disease will be referred to appropriate ophthalmological care in a health facility (the district hospital) or the any other health facility in Ghana, where affected subjects will obtain appropriate care (University hospital or private ophthalmologist). <u>All ocular disease exclusion criteria apply to either eye. Bilateral disease is not necessary to exclude a participant. A participant will be excluded if any of the criteria are met for one eye.</u>
    a. Any cataract of any type preventing clear visualization of fundus or imaging on Optical Coherence Tomography (OCT).
    b. Severe retinal nerve fiber layer thinning in the superior and inferior quadrant analysis on Ocular Coherence Tomography of the optic nerve with a corresponding visual field defect of grade 2 or worse on the same eye.
        i. If Ocular Coherence Tomography is not available, the following exclusion criteria will apply: vertical Cup/disc ratio on fundoscopy (not by OCT reading) greater than or equal to 0.80.
    c. Intraocular pressure (IOP) greater than or equal to 25 by Goldmann tonometry .[12]
    d. Retinal Detachment or Retinal Break
    e. Acute ocular infection (i.e., Viral conjunctivitis, corneal ulcer, endophthalmitis)
    f. Optic Atrophy with visual field defect reproducible on confrontation visual field testing..
    g. Exam consistent with Herpes Simplex Virus eye infection
    h. Homonymous hemianopsia, quadrantopsia, bitemporal hemianopsia, or central scotoma related to cerebral vascular disease by Automated Visual Field testing and confrontation visual field testing.
    i. Acute Angle Closure Glaucoma
    j. Gonioscopy grade 0 (slit) limiting ability to safely dilate patient
    k. Severe Tremor, blepharospasm, or other voluntary or involuntary motor condition that prevents ability to examine patient with slit lamp, OCT, gonioscopy, IOP measurement, fundus photography, and Frequency doubling technology perimetry.
    l. Cognitive impairment sufficient to prevent ability to understand and perform Visual Acuity Test with Tumbling E chart, confrontation visual field, slit lamp exam, or any other ocular exam component.
    m. Optic nerve edema
    n. Active retinopathy or retinitis not attributable to onchocercal disease
    o. History of uveitis not associated with onchocercal disease
    p. Any pre-existing chorioretinal scar or retinal degeneration and other significant retinal pathologies (foveomacular schisis, dystrophies, arterial macroaneurysms etc) involving the macula.
    q. Severe ocular pain, that patient rates as 9 or 10 out of 10 pain.
    r. Best corrected or pinhole visual acuity worse than 6/60 (20/200)
    s. Age related macular degeneration (AMD)
3. Significant comorbidities such as renal insufficiency, liver failure, or any other acute or chronic illness identified by study clinicians and investigators that interferes with the participant's ability to go to school or work or perform routine household chores.

4. Prior allergic / hypersensitivity reactions or intolerance to IVM, albendazole, or DEC.
5. Treatment with IVM outside of the study after the pre-treatment clearing dose provided in the Part I study.
6. >5 motile Mf in the anterior chamber in either eye at the time of enrollment (after pre-treatment with IVM).

   Note: The cut-off of 5 Mf was suggested by external reviewers of our proposal to the Gates Foundation. These are experts in onchocerciasis selected by the Foundation. The reviews were anonymous, so we do not know their names. They also suggested that we exclude persons with any Mf in the posterior segment of the eye, and we have added that exclusion criteria that in the protocol.
7. Any Mf identified in the posterior segment of the eye at the time of enrollment (six months after pre-treatment with IVM).
8. Any other condition identified by study clinicians or investigators that may preclude participation in the study.

4.3 Participant Exclusion from Routine MDA

Participants will be informed that they should not participate in MDA distributed by the Ghana Health Service in their communities during this study period. Participants will receive a laminated card to identify them as participants in the study (Appendix A). Participants will present these cards to community drug distributors. Study investigators will communicate with local health officials responsible for MDA to inform them of the study and ensure that they are aware that there are study participants who should be excluded from routine MDA for the duration of the study.

4.4 Randomization

At least six months after treatment with ivermectin (-5.5 months or +12 months) in the preceding Part I trial, participants will first be split into two strata – those *without* ocular Mf detected six months after ivermectin pretreatment in the Part I preceding study *AND* *without* ocular Mf detected at baseline in the part II study will be in stratum 1. Those participants *with* ocular Mf detected 6 months after ivermectin pretreatment in the preceding study *OR* *with* ocular Mf detected at the baseline exam for this study will be in stratum 2. Stratum 1 will be enrolled first, followed by stratum 2. This is a safety precaution that will ensure that participants with *any* Mf in either eye 6 months after ivermectin pretreatment in the previous study will be the last to be dosed with combination drug treatments in this study. The decision to stratify so that persons who might be at higher risk are dosed last was based on recommendations from external expert reviewers of our grant proposal.

Members of each stratum will be evenly randomized into one of three treatment arms:

Stratum 1: Ocular Mf negative by slit lamp six months after ivermectin pretreatment (in Part I) **AND** ocular Mf negative by slit lamp at baseline enrollment

1. IVM + ALB - Single dose of oral IVM (150 µg/kg) plus ALB (400 mg) (IVM/ALB)
2. IDA x 1 dose - Single dose of oral IVM (150 µg/kg), DEC (6 mg/kg) and ALB (400 mg)
3. IDA x 3 doses -Once daily for 3 days oral IVM (150 µg/kg), DEC (6 mg/kg) and ALB (400 mg)

Stratum 2: Oular Mf positive 6 months after ivermectin pre-treatment (in Part I) ***OR*** ocular Mf positive at baseline enrollment (ocular Mf counts by slit lamp greater than 0 but < 5 in either eye)

1. IVM + ALB - Single dose of oral IVM (150 µg/kg) plus ALB (400 mg) (IVM/ALB)
2. IDA x 1 dose - Single dose of oral IVM (150 µg/kg), DEC (6 mg/kg) and ALB (400 mg)
3. IDA x 3 doses -Once daily for 3 days oral IVM (150 µg/kg), DEC (6 mg/kg) and ALB (400 mg)

4.5 Treatment masking

While this is an open label study and there is no placebo treatment group, all efforts will be made to ensure that that medical/technical staff assessing skin Mf, AEs and ophthalmological findings will be unaware of initial baseline skin and ocular Mf findings and treatment arm as best as possible. This is especially important for those performing AE assessments and for persons reviewing nodule slides, because the primary objectives depend on those evaluations. A study pharmacist will be responsible for managing the randomization assignment list and treating patients accordingly. The pharmacist should be the only person at the study site who will know treatment assignments. Treatment information will be kept separate from the rest of the participant case report form and shared only if required for medical purposes. Efforts will also be made to assure baseline infection status is not shared with examiners unless required for medical purposes in the case of severe or serious AEs.

The histologists reading the nodule histology slides will only be provided with histology slides identified by a patient number and nodule number. The histologist will have no other information about the patient.

Treatment and baseline infection status will be available to the study sponsor, data analysts, and DSMB. The data manager will un-mask the treatment assignment after the last participant has had their 3 month post-treatment evaluation. The safety data will then be compared by study arm. That will complete the safety study, which is separate from the efficacy study. Persons evaluating nodules will be masked regarding Mf counts and treatment assignment to avoid bias. Nodule data will be analyzed after the nodule evaluations have been completed. That part of the study is focused on the issue of whether IDA is superior to IVM and albendazole alone for killing or sterilizing adult worms.

4.6 Study Procedures

The six-month follow-up parasitological, general health, skin, and ocular examination from the preceding Part I study will serve as the baseline for this Part II study. Participants will be housed in dormitory-like wards of the University of Health and Allied Sciences School of Public Health Research Centre on the Hohoe Municipal Hospital grounds for 7 days following treatment with medical supervision. Participants will be fed three times daily. Accommodation, feeding, laundry and entertainment will be provided at no cost to participants.

Participants will be dosed with study medications in the dormitory-like wards of the University of Health and Allied Sciences School of Public Health Research Centre on the Hohoe Municipal Hospital grounds. Medications will be administered by Study Pharmacist assisted by the study head nurse.

After treatment participants will be examined daily in the center for any adverse events for 7 days before being discharged to their communities. Full ocular examinations will be performed before treatment, on day 7 and at 3 months after treatment. Physicians will also obtain medical histories and perform physical examinations before treatment and on day 7 after treatment. In addition, any participant who reports any change in vision or ocular symptoms between day 7 and 3 months will be transported back to the clinical facility for a full ocular examination. Participants will be told to report to the study village coordinator if they develop any symptoms. In the event that some individuals fail to clear ocular Mf after IVM pretreatment (see Table 1), they will still be eligible for enrollment in the study if the number of motile Mf in the anterior chamber of each eye is ≤ 5. Individuals with >5 motile Mf in the anterior segment or any Mf observed in the posterior segment of the eye will be excluded from the study, and they will be referred for routine treatment with IVM, and adult worms will be assessed by standard histological methods for viability and fertility.

Samples will be collected for later studies of biomarkers of *Onchocerca* infection. 5ml of peripheral venous blood and 10 ml of first void morning urine will be obtained prior to the Part II treatment and at 3, 12, and 18 months after treatment. Post-treatment samples will be compared with samples collected prior to the Part I IVM clearing dose and with samples collected just prior to treatment in this study (Part II). Samples will be stored at the UHAS study site until they are shipped to the USA for analysis (see paragraph 6.1). Analysis of samples will take place at the Weil Lab at Washington University in St. Louis. Samples will be stored for up to 35 years and then destroyed according to regulatory guidelines.

Parasitological Evaluations

Palpation for subcutaneous *Onchocerca* nodules: This baseline information will be available from the preceding related study, but nodule palpation will also be performed at the time of nodulectomy

Skin snip testing to detect microfilaria in skin

Skin snip testing for Mf will be performed at 3, 12 and 18 months. We will obtain four (4) skin snips using a corneoscleral punch. Each snip will be weighed on an analytical balance and incubated for at least 8 hours in isotonic saline in a well of a flat-bottomed micro-titre plate at ambient temperature. Microfilariae will be counted by microscopy. Mf number and skin snip weight will be recorded. The mean skin microfilarial density will be calculated and recorded as mf/mg. Skin snips will also be preserved for later PCR testing to detect parasite DNA (more sensitive than microscopy).

Nodulectomies

Nodulectomies will be performed at 18 months after IDA treatment. Nodules will be surgically excised by Ghanaian study medical officers, under local anesthesia (1% xylocaine) in a specially designated room at the University of Health and Allied Sciences School of Public Health Research Centre using aseptic procedures. At the final visit at 18 months post-treatment participants will be brought to the centre in Hohoe for 2 days for nodulectomies. After the procedure they will then be returned to their communities where a study nurse and will be stationed to continue wound management.

Wound treatment for Nodulectomy

Wound management will begin with the nodulectomy incision and continued till all wounds are healed. Subjects will usually be detained on the ward after surgery for two days or longer. During this period, wounds are examined for bleeding, hematoma formation and dehiscence. Subjects will advised to stay off work for the first week post surgery to assist in wound healing. Compensation will be paid for loss of earnings for the seven (7) days if appropriate. The first change of dressing is done on the first day after nodulectomy. On day 10 post-nodulectomy, sutures will be removed. Subsequent dressing changes will occur on alternate days until wound is completely healed. Nurses will assist with wound care management in the community. This study draws participants from 5 communities, but at any one time there will only be participants from 1 or 2 communities who require care for nodulectomy wounds. Additionally, cohorts of participants receiving nodulectomies are staggered so that there are not more than 20 participants requiring wound care at one time. Two nurses will be available in the communities to monitor wound healing and provide wound management and dressing changes as needed.

Ocular Examinations:

The following **ocular examinations** will be performed by qualified medical personnel at the University of Health and Allied Sciences School of Public Health Research Centre in Hohoe:

1. Visual acuity exam: using a standard Snellen or E chart, individuals will stand or sit 20 feet (6 meters) from the chart with both eyes open. They will be asked to cover one eye with the palm of the hand (or a small paddle) and read out loud the smallest line of letters that can be seen. The test is done in each eye, one at a time.
2. Visual fields: We will assess visual fields either by confrontational testing and/or by automated VF perimetry testing. Both tests are widely used and non-invasive.
3. Pupillary reflex response testing: This will involve shining a light in one eye and then examine pupillary response and the degree of constriction in the illuminated pupil as well and consensual response, or the response in the opposite pupil. This test can suggest asymmetric optic nerve or retinal disease.
4. Applanation tonometry: this test measures intraocular pressure. We will apply local anesthetic drops approved for use in Ghana in each eye to numb the surface of the eye and then apply a fine fluorescein strip to tear film of each eye. The slit lamp is placed in front of the individual and the Goldmann tonometer will measure the intraocular pressure.
5. Slit lamp examination: using a slit lamp, we will evaluate each participant for Mf in the anterior chamber of eye (after having the subject position their face downwards for at least 2 minutes). We will look for the presence of anterior segment and posterior segment disease, including but not limited to uveitis, limbitis, cataracts, corneal scarring, optic nerve disease, and retinal disease. A 90 Diopter and 78 Diopter lens will be used in conjunction with the slit lamp to visualize the fundus (fundoscopy).
6. Indirect ophthalmoscopy: this fundoscopy examination technique will be optionally performed at the discretion of study ophthalmologists and optometrists to evaluate the fundus of the eye (posterior segment) particularly for suspected retinal detachment Prior to the test, the examiner will apply eye drops approved for use in Ghana to dilate the pupil and then use the indirect ophthalmoscope to visualize the different structures in the posterior segment of the eye after the pupil is dilated.

7. Retinal photography: Flash photography of the retina and optic nerve will be taken to document anatomical findings. Black and white and color images will be recorded. Retinal photography will be taken with the pupils dilated. Pupils will already have been dilated for fundoscopy (see above), and therefore, retinal photography will not require re-dilation of the pupil. Faces will not be included in these photographs**.**
8. Slit-lamp Photography. Photography of the anterior chamber will be taken whenever possible to record presence of microfilariae and anterior segment findings. Short videos will also be taken to be record microfilarial motility or immotility. This can be performed prior to dilation, and will not require re-dilation of the pupil. Faces will not be included in these photographs.
9. Optical coherence tomography (OCT): this is a non-invasive method that uses low intensity laser light to capture micrometer-resolution, three-dimensional images of both the anterior and posterior segment of eye. It can visualize the full thickness cross-section of cornea, conjunctiva, anterior chamber, iris and retina and optic nerve head. Prior to the test we will apply eye drops (1% tropicamide) to dilate the pupil, which will already have been dilated for fundoscopy. Then the subject will be positioned in front of the OCT scan, resting the head on a support to keep it motionless. The equipment scans the eyes without touching them. Scanning takes 5-10 minutes. Because this technology utilizes light waves, media opacities such as vitreous hemorrhages, dense cataracts or corneal opacities may interfere with an optimal imaging.

General health examination and skin evaluation.

Each participant will undergo a routine history and physical examination as part of the screening process for eligibility that will include vital signs, height, and weight. We will also collect history of any past and current medications with a focus on onchocerciasis related skin and/or eye disease and any prior treatment for onchocerciasis over the past 6 months

Skin evaluations will include a careful examination of the skin by a study physician; significant abnormalities (grade 2 or higher) present during the baseline examination will be documented by photography. Skin survey and records of baseline skin disease with photographs in persons with significant abnormalities will be collected in the case report form. **Faces will not be included in photographs.**

4.7 Participant Treatment

At baseline, participants will be given ivermectin (150 µg/kg) pretreatment. This pretreatment will be provided the participants' villages near their homes following their consenting to participate in the study. *Please note, *all participants* will be treated with ivermectin at baseline to ensure that they have low skin and ocular Mf counts at the time of enrollment. (This treatment is *in addition to* the pretreatment with ivermectin given in the Part I study).

After the baseline treatment with ivermectin alone, participants will be randomly assigned to one of the following treatment arms (all oral medications)*:

I. **Ivermectin 150µg/kg plus albendazole (400 mg)**

II. IDA single oral dose

   a. Ivermectin, 150µg/kg

b. Diethylcarbamazine citrate, 6 mg/kg

c. Albendazole, 400 mg

III. IDA three one time daily oral doses

a. Ivermectin, 150µg/kg

b. Diethylcarbamazine citrate, 6 mg/kg

c. Albendazole, 400 mg

Randomized treatment to one of the three treatment arms will occur in cohorts of up to 20 individuals. This is based on the project's capacity for transport, examination, and housing of participants. Initial participants will be individuals who had zero Mf in the eyes six months after pre-treatment with IVM in Part I and zero Mf in the eyes at baseline for Part II. If IDA proves safe in all of these participants,(i.e. if there are no serious or severe adverse events in *any* participant in the first 7 days) the study will continue with participants who had Mf in the anterior chamber in either eye six months after IVM pre-treatment in the Part I study or at the baseline exams for this study. Study investigators will monitor the safety of the participants in each treatment cohort for 7 days before dosing the next cohort, and they will suspend the study if any treatment-related serious or severe adverse events occur. If SAEs are reported, the DSMB will be asked to review the cases and to implement study stopping and pausing guidelines if the SAEs are found to be related to study treatments.

4.7.1 Plan for Re-dosing

Participants who vomit within 30 minutes after dosing will be redosed the same day at least two hours after the first attempt. If they vomit the second dose, they will be excluded from the study and provided treatment with ivermectin alone the next day.

4.7.2 Concomitant Medications

Participants will be told to continue to take their regular medications. Additionally, each participant will be instructed and provided a study ID card during consenting that indicates that they are participants in a clinical trial and should not take ivermectin/Mectizan MDA given by mass drug administration (MDA) program or other groups until nodulectomies have been performed at 18 months.. Each participant will be instructed and provided a study ID card during consenting that indicates that they are participants in a clinical trial and should not take ivermectin (or Mectizan) provided by a government program or other group until nodulectomies have been performed 18 months after the Part II treatment.

4.8 Assessment of Adult Filarial Worms

Some of the primary and some secondary efficacy endpoints for this will be based on histology results from examination of nodules collected from participants at the end of the study. Nodulectomies will be performed at UHAS School of Public Health Research Centre in Hohoe. The nodules will be preserved before being sent to a histology laboratory (location to be decided) for embedding, slide preparation, and histological staining. This will include H&E for general morphology, APR-immunostain for differentiating dead and life worms[43], and Gomori iron stain for

worm age assessment. The stained nodule sections will be read by two independent readers. The readers will not have access to each other's results until after they have entered their results. The two results will be compared, and the two readers will work together to reconcile any discrepancies. A third reader can be used to reconcile discrepancies if the two readers cannot agree. The reconciled result will be considered to be the final result.

4.9 Study Duration and Timeline

Participants will be followed for 18 months after treatment. Participants will be transported to the study site at the University of Health and Allied Sciences (UHAS) School of Public Health Research Centre in Hohoe, Ghana to complete enrollment and treatment procedures and for assessment of adverse events. Participants will be housed at a clinical facility on the municipal hospital grounds for 7 days after treatment. Participants will be brought back to the clinical research facility in Hohoe 3 months after treatment for 2 days in order to complete ocular re-examinations and skin snip testing. Skin snips will be collected 12 months after treatment in the participants' home village. The final visit will occur at 18 months ±60 days when participants are once again brought to the clinical facility in Hohoe for 2 days to have skin snip testing and nodulectomies.

The following tolerances will be allowed for flexibility in scheduling participant visits: Day 7 visit +/- 1 day. Month 3 visit +/- 14 days. Month 12 +/-30 days. Month 16 +/- 60 days.

The on-study period per participant is approximately 20 months and it is anticipated that the duration of the study will be up to 24 months, including 4 months for recruitment.

4.10 Investigational Product Supply and Storage

Drugs used for this protocol will be purchased from manufacturers who donate their product for use in the Global Program to Eliminate Lymphatic Filariasis. Alternatively, WHO approved generic versions may be purchased. If necessary, import approvals will be requested from the government of Ghana.

Albendazole and IVM are approved for use and widely used in Ghana for lymphatic filariasis and/or onchocerciasis control and elimination. However, DEC is a not approved for use in people with lymphatic filariasis or onchocerciasis. Thus use of DEC in this study will require approval by ethical review committees and by the Ghana FDA.

Detailed information for each drug is available from the manufacturers and the package inserts are included an an appendix to this protocol (Appendix B).

Investigational products should stored in a secure, locked pharmacy room. In this study all drugs will be stored at no greater than 30°C per the package insert. Documentation of tempature logs will be done per the study site SOPs.

5 POTENTIAL RISKS AND BENEFITS

5.1 Risks of Skin Snips:

This procedure is associated with a small risk of bleeding and infection. The skin will be disinfected before a snip is taken with a sterile scleral punch. The snip (about 2 mg) will cause a superficial wound of the skin at the iliac crests and calves, which will be covered with a plaster for one day. These procedures are routine and designed to minimize risks of infection or bleeding. The scar will be 2 mm or less in size.

5.2 Risk of ocular exams

The ophthalmological procedures are routine and associated with minimal risk. The possible risks are related to the ocular medications administered during these tests. To perform applanation tonometry and slit lamp examinations investigators will apply local anesthetic eye drops approved for use in Ghana (e.g lidocaine, proparacaine or tetracaine), which may cause mild local reactions such as conjunctival hyperemia and ocular irritation/burning.

To perform indirect ophthalmoscopy, retinal photography and OCT, investigators will apply eye drops to dilate the pupil (e.g. generic tropicamide) which may cause transient blurred vision, photophobia, ocular stinging. These eye drops are routinely used in persons undergoing ophthalmology examinations. Dilation eye drops will only need to be applied once, and the pupils will remain dilated for the remainder of the examination. Rare adverse events include increases in intraocular pressure and superficial punctuate keratitis; other uncommon systemic side effects include xerostomia, tachycardia, headache, nauseas and vomiting.

5.3 Risk of study medications:

The combinations of IVM plus albendazole or DEC plus albendazole are widely used in MDA programs. There also have been 3 clinical trials and extensive safety trials of IDA in 5 countries. A published study demonstrated no significant interactions between the three IDA drugs[35]. The WHO has recently recommended use of IDA for LF elimination programs in areas without co-endemic onchocerciasis or loiasis that are not currently on track to meet the 2020 deadline (http://www.who.int/neglected_diseases/news/WHO_recommends_triple_medicine_therapy_for_LF_elimination/en/). Potential risks for each drug separately, with some indication of how likely these are to occur, are summarized below.

Risks of Diethylcarbamazine (DEC): This drug is known to be safe for treatment of LF and hundreds of millions of people are treated each year by MDA programs in areas without coendemic onchocerciasis or loiasis. Mild to moderate adverse events are seen in persons with microfilaremia. The most adverse events are headache, fever, joint pain, unusual tiredness or weakness. Less common are dizziness, nausea or vomiting. Persons with onchocerciasis often experience pruitis and dermal edema. Ocular AEs in onchocerciasis patients that have not received a clearing dose of IVM are described above (see *Background*).

Risks of Albendazole (ALB): This medication is safe and is provided as MDA to hundreds of millions of persons in Africa each year by programs to eliminate LF and onchocerciasis and to children in school-based deworming programs. Headache, nausea, stomach pain and/or vomiting sometimes occur after treatment, and this tends to be more common in persons with heavy STH infections. Severe allergic reactions occur rarely, and these can include rash, hives, itching, difficulty breathing, tightness in the chest, swelling of the mouth, face, lips, or tongue. Stevens-Johnson syndrome has been reported, but this is very rare. Mild, transient elevation in liver transaminases rarely occurs after single dose ALB treatment. More severe liver toxicity and leukopenia sometimes occur in persons treated with long courses of ALB with doses of 800 mg per day or more.

Risks of Ivermectin (IVM): This medication is safe and is provided as MDA to more than 100 million persons in Africa each year by programs to eliminate LF and onchocerciasis. The most common side effects are diarrhea, dizziness and nausea. Persons with onchocerciasis sometimes experience rash, hives, itching (see Mazzotti reactions, below), difficulty breathing, tightness in the chest, swelling of the mouth, face, lips, or tongue; eye pain, swelling, or redness. Seizures, fainting, mild decrease in leukocyte counts, elevated liver function tests, and cardiovascular effects that included tachycardia and orthostatic hypotension have been reported, but these adverse events are rare and not necessarily related to IVM treatment.

Mazzotti Reactions: These AEs are caused by inflammatory and immune reactions to microfilariae that are killed by IVM or DEC as described above. Mazzotti reactions need to be differentiated from any coincidental illnesses that may occur in temporal relationship with IVM treatment. Common systemic clinical manifestations of the Mazzotti reaction include pruritus, rash, lymphadenitis, headache, myalgia, arthralgia, hypotension, fever, and swelling of the face and limbs. Ocular events include epiphora, photophobia, and conjunctival injection. The factors that govern the Mazzotti reaction include the intensity of infection, the dose regimen, and the microfilaricide used. These reactions usually resolve spontaneously, although treatment with ordinary analgesics (e.g. acetaminophen; aspirin,

ibuprofen) and antihistamines (e.g. diphenhydramine) are often provided in clinical trials. Most AEs resolve within 3- 4 days after treatment.

The DEC is manufactured by Pfizer and will purchased through Amtrex Enterprises. Ivermectin and albendazole will be obtained from Merck and GlaxoSmithKline respectively. Each drug purchased, will have certificates of analysis which will be retained in the study trial master files and will be provided to the Ghana Health Service and FDA if required**.**

5.4 Risks of nodulectomy

Nodulectomies are a standard procedure to treat onchocerciasis in endemic countries since current approved medications cannot kill the adult worms in nodules. Nodulectomies will be performed in an operating room under sterile conditions. Onchocercomata will be removed under local anesthesia by an experienced physician. There is a risk of blood loss or infection at the incision site however, careful sterile techniques should mitigate this risk. Subjects will be required to stay from normal work for 1 week after nodulectomy to facilitate wound healing and will be paid for their loss of earnings.

5.5 Conducting the trial during the Covid-19 pandemic

Potential Risks to Participants

Severe acute respiratory syndrome coronavirus 2 (SARS-CoV-2 also known as COVID-19) is a virus that was first reported in China in late December 2019, but has since spread to most countries in the world, including the presence of some cases in Ghana. COVID-19 is spread from person-to-person contact through respiratory droplets of an infected individual (eg. from coughing, sneezing, breathing). In the current clinical trial, patients will be in contact with other patients and staff during transport to the study site and during their stay at the health center for enrollment and follow-up exams.

Mitigation Procedures

The following measures will be put in place during the trial to prevent the transmission of SARS-CoV-2:

- **The Research team will follow the Ghana Health Service Ethics Review Committee (GHS-ERC) Guidelines for conducting research during the COVID-19 pandemic**
- **Participants will be educated on the COVID-19 pandemic with the emphasis on how COVID-19 is now being transmitted from human to human, adequate protection by practicing good personal hygiene, maintaining physical distancing, hand washing with soap and running water, and the use sanitizers and wearing of facemasks.**
- **Participants will be triaged in their communities by asking whether they have symptoms of cough, catarrh, and/or fever and will also have their temperatures checked. All participants with suspected symptoms of COVID-19 will be referred to the Nkwanta South District COVID-19 team.**

- **Participants who leave the study site after a visit will be encouraged to share any covid-19 test that they have been made to take as soon as they do so and the results of the test as soon as they become available.**
- **Physical distancing will be maintained in project vehicles during transportation to and from participants' communities and participants will be required to wash their hands with soap and water, sanitise their hands with alcohol-based sanitisers and wear facemasks during all transportations.**
- **On arrival at the center, participants will be required to wash their hands with soap and running water and to sanitise their hands with alcohol based sanitisers.**
- **To ensure proper physical distancing, fewer participants will be housed in the center at one time (no more than 14 instead of 20).**
- **The researchers will follow all preventive protocols as advised by WHO and the national directives on COVID-19 including adequate protection by practicing good personal hygiene through regular hand washing with soap and running water, the use of sanitizers, protective gloves and wearing of facemasks. Social/physical distancing will be observed at all times.**
- **Staff and participants will wear facemasks in examination rooms. Hand sanitizer will be in the exam rooms, and staff will sanitize all instruments such as slit lamps and OCT that contact patient's skin after each examination.**

5.6 Study Benefits

Benefits to Individuals

All participants in the study have onchocerciasis, and participants will benefit from the study treatments. Treatment with IDA may sterilize or kill adult worms better than treatment with the comparator regimen IVM/ALB. Participants will also benefit by having onchocercal nodules surgically removed. Drugs used in this study (ALB and IVM) are also active against intestinal nematode worms.

Public health benefit

If IDA is more effective for achieving sustain clearance of Mf than the comparator (IVM/ALB), this could lead to accelerated elimination of onchocerciasis in many areas in Africa. If IDA (after IVM pretreatment) is well tolerated in persons with onchocerciasis, this strategy might be used to accelerate LF elimination in Africa in areas with co-endemic LF and onchocerciasis even if the regimen is not effective for onchocerciasis. However, additional larger safety studies would be needed before I/IDA could be accepted as a MDA regimen for use in areas with onchocerciasis.
Cost to Subjects

There will be no cost to study participants, and study medications will be provided free of charge. All costs related to parasitological, ophthalmological and clinical monitoring and nodulectomies will be covered by the study. Study participants will be provided free transportation to Hohoe and back to their home communities, and they will be provided with food and accommodation in Hohoe. Participants will be visited and examined for follow-ups in their communities by the research team. All visits and examinations including wound dressings will free to participants.

5.7 Study Compensation

Compensation for Loss of Earnings

Subjects will not be paid for participation in this study. Subjects will be compensated for the loss of earnings for the participating in the study. The compensation will be based on the number of days spent participating in protocol related activity at the village/clinic facility in Hohoe. For each day of participation, each participant will be compensated forty Ghana Cedis (GH₵ 40:00) per day. Minors participating in the study will be compensated at the same rate. Compensation will be paid at the end of enrollment and at the conclusion of each follow-up visit**.**

Compensation for Research Related Injuries

Commercial clinical trial insurance will be purchased for this study. In the event that a subject experiences an AE attributable to treatment, the project will help support any medical treatment and hospitalization required. Participants (or their families) will be compensated if any participant dies or is disabled because of participation in the study according to procedures followed for clinical research projects in Ghana.

The study will comply with Ghanaian government guidelines that require insurance to cover all participants, investigators and the institution in the clinical trial in case of damage to health due to participation in the clinical trial. The risk for the current trial is very low since the study medications are widely used and considered to be safe. The clinical evaluation procedures (skin snips, ophthalmological examinations, and nodulectomies) are standard of care, low risk procedures that are unlikely to damage health.

6 INSTRUCTIONS FOR SPECIMEN PREPARATION, HANDLING, AND STORAGE

6.1 Specimen and Sample Handling

Specimens will be tested in clinical laboratories at Kpassa (skin snips) and at the UHAS School of Public Health Research Centre in Hohoe, Ghana (blood, urine, skin snips). Onchocercal nodules will be fixed in preservative and processed for histology at a histology laboratory with CAP (College of Amercian Pathologists) or an equivalent international certification.

Samples (serum, urine, skin snips, nodules) sent outside of Ghana for testing or storage will be completely de-identified (study ID number only). De-identified samples will be sent to Washington University in St. Louis and/or Case Western Reserve University (USA). Samples may be stored at one or more of these sites. These samples will be used for onchocerciasis related studies only.

6.2 Specimen Shipment

Nodule histology slides will be read in two independent sites. One site will be the DOLF project reference laboratory (Weil Lab) at the Washington University in St. Louis. Shipments of nodules and slides ill comply with Ghanaian and United States federal rules and with IATA regulations. The Weil Lab at Washington University in St. Louis is located at 4444 Forest Park Blvd, St. Louis MO

63110. Samples will be stored for up to 35 years and then destroyed according to regulatory guidance.

7 SAFETY REPORTING AND SAFETY MONITORING

Most treatment related adverse events (cutaneous or ocular) result from the death of microfilariae in the skin and/or the eye are related to death of parasites and are not due to direct toxic effects of the drugs in humans. Other adverse events such as nausea and abdominal pain sometimes occur after treatment with IVM, ALB, or DEC. These may be related to local physical effects of the tablets or to effects of these drugs on intestinal worms. AEs related to death of *O. volvulus* Mf typically occur following the initial (first time) treatment when the burden of Mf in the skin and eye is greatest. These AEs typically start 1 or 2 days after treatment and they may occur up to 7 days after treatment. After treatment, participants will be assessed daily in the center for adverse events for 7 days before being discharged to their communities.

If participants develop severe AEs (grade 3, events that interfere with activities of daily living) or serious AEs for any reason after they return to the Kpassa area, they will be transported to Kpassa Health Centre, Nkwanta District Hospital, or UHAS School of Public Health Research Centre at Hohoe hospital for evaluation and management. SAEs will be reported to the Ghana Food and Drug Administration (FDA), UHAS Research Ethics Committee, Ghana Health Service Ethics Review Committee (GHS-ERC), to manufacturers of study medications, and to other institutional review boards per Good Clinical Practice (GCP) guidelines and timelines.

More detail on Safety Reporting can be found in the Safety Plan corresponding to this study.

7.1 Definitions

Adverse Events (AEs)

Adverse events (AEs) are any untoward medical occurrence in a clinical investigation participant who has received a study product intervention. AEs do not necessarily have to be causally related to treatment with the study product. An AE can, therefore, be any unfavorable and unintended symptom, sign (including an abnormal laboratory finding) or other disease temporally associated with the use of a study medicinal product, whether or not the AE is considered to be related to the study medicinal product.

An AE does not include:

- Medical or surgical procedures (e.g. surgery, tooth extraction, transfusion). The condition that leads to the procedure is an adverse event
- Pre-existing diseases or conditions or laboratory abnormalities present or detected prior to the screening visit that do not worsen.

Ocular Adverse Events (OAEs)

Ocular adverse events are adverse events in the Guide for Assigning Adverse Events, or any other adverse event pertaining to the eye resulting in symptomatic, visual or functional disturbance.

Serious Adverse Event (SAE)

Any adverse event that results in any of the following outcomes:

- Death
- Life-threatening (immediate risk of death)
- Inpatient hospitalization or prolongation of existing hospitalization
- Persistent or significant disability or incapacity
- Congenital anomaly/birth defect
- Important medical events that may not result in death, be life threatening, or require hospitalization may be considered a serious adverse event when, based upon appropriate medical judgment, they may jeopardize the participant and may require medical or surgical intervention to prevent one of the outcomes listed in this definition. Examples of such medical events include allergic bronchospasm requiring intensive treatment in an emergency room or at home, blood dyscrasias or convulsions that do not result in inpatient hospitalization, or the development of drug dependency or drug abuse.

Ocular SAEs

An ocular severe adverse event will be defined as:

- An AE causing severe visual loss, defined as a decrease in VA of > or = 6 lines compared with baseline VA or a decrease in VA to level of 6/60 or worse
- An AE requiring medical or surgical intervention to prevent permanent loss of vision;
- An AE associated with severe intraocular inflammation;
- Conjunctival or scleral necrosis
- Corneal Edema or persistent corneal erosion
- Retinal Detachment
- Retinal Hemorrhage
- Optic Atrophy
- Increased IOP > 15 mmHg from baseline

Unexpected

An adverse reaction, the nature or severity of which is not consistent with the applicable product information (e.g., Package Insert).

Expedited Safety Report

Documentation in appropriate form and format summarizing an SAE that meets expedited safety reporting criteria, submitted within the required reporting time frame of applicable regulatory authorities and/or institutional review boards (IRBs) or ERCs of participating countries.

7.2 Assessment of Adverse Events

Adverse event monitoring will be performed daily for 7 days following treatment with study medications. Participants will be kept at a the *UHAS School of Public Health Research Centre* clinical facility at the Hohoe municipal Hospital for 7 days after treatment so that they can be

closely monitored for adverse events and provided care if needed. See Appendix C for a sample AE assessment form.

Adverse events will be defined using a modified Common Terminology Criteria for Adverse Events (CTCAE) grading table. Study ophthalmologists have added additional detail to the CTCAE tables for potential ocular adverse events related to this study (Appendix D).

Evaluations will be documented on the case report forms (either paper or entered directly into an electronic CRF using a smart telephone or tablet computer).

After 7 days participants will be returned to their homes. They will be instructed to contact the study village coordinators who are resident in their communities to inform the study investigators of any late adverse events and arrange transportation to the Kpassa Health Centre/Nkwanta District Hospital/ or *UHAS School of Public Health Research Centre* at Hohoe hospital medical care if required.

7.3 Serious Adverse Event (SAE) Assessment and Management

Project ophthalmologists will manage any significant ocular AEs with treatments such as topical or periocular injections of anti-inflammatory medications (e.g., steroids). Extra-ocular AEs such as Mazzotti reactions (fever with skin rash, pruritus, and malaise) will be managed by study personnel with medications such as anti-pyretics, anti-inflammatory drugs and anti-histamines, which are routine, approved medications and will be prescribed by the study clinician. Study participants with definite or suspected SAEs (any event ≥ grade 3) will be referred to a physician or appropriate health care professional for evaluation. The physician will determine whether the event qualifies as a serious adverse event (SAE).

All SAEs will be reported to regulatory authorities and to the Ghana FDA within the stipulated timelimes (see Safety Plan). The report will specify whether the SAE was related, possibly related or unrelated to treatment and also specify whether the type of AE observed is unexpected based on the study drug's package insert. (See Appendix C for a sample SAE report form.)

All AEs and SAEs will be recorded in source documents. All AEs and SAEs will be recorded in the CRFs approved by GHS-ERC. SAEs and follow-up information will be reported to each IRB per their approved reporting policies.

7.4 Reporting of Pregnancy

Pregnancy is an exclusion criteria for this study. Although not AEs, pregnancies are reportable events. If a pregnant woman is inadvertently dosed, efforts will be made to follow-up and report the outcome of the pregnancy (e.g., healthy baby, miscarriage, etc.) Any pregnant woman inadvertently dosed who has a miscarriage or spontaneous abortion will be reported as an SAE. If a participant becomes pregnant after treatment they may remain in the study, but the study will not actively follow the outcome of the pregnancy. Pregnancy will be diagnosed by a urine human chorionic gonadotropin (urine HCG) test. If a pregnant woman is inadvertently dosed with study medications for either intervention arm, the baby delivered as a result of that pregnancy will be followed for one year after birth.

7.5 Data Safety Monitoring Board

A Data Safety Monitoring Board (DSMB) will be formed consisting of at least 4 experts including: at least 2 physicians, one of which is an ophthalmologist; as well as one statistician, and at least one scientist or physician who is knowledgeable in onchocerciasis and neglected tropical diseases. At least one member of the DSMB will be a Ghanaian scientist or physician. Study investigators will send safety reports to the DSMB at least monthly, and the DSMB will meet when the first 30 participants have been enrolled, at the midpoint of the study, and after the second stratum of participants is complete. Thereafter, meetings will take place quarterly until all participants have completed 3 month follow-ups. Ad hoc meetings will be held as needed and in the event that the PI or other study staff note safety concerns during enrollment. The DSMB will monitor the type and frequency of AEs and SAEs recorded by the teams and provide guidance to the PIs and the teams in the field. A draft of the DSMB charter is located in Appendix E. The final version of this charter will be submitted to all IRBs after members of the DSMB have confirmed their participation.

7.6 Interim Analyses

Data will be analyzed during the study for "go" or "no go" decisions for continuation of the study. Interim analyses will be performed after enrollment and 7 day follow-up is completed for participants in stratum 1 and then again at the end of stratum 2. A third interim analysis will be performed after the 3 month assessments have been completed for all participants. Among other things, the DSMB should consider whether severe AEs are significantly more common after IDA than after IVM/ALB treatment. The DSMB will review safety data on a regular basis, and they will review all severe or serious ocular AEs as and when they occur. The DSMB will consider stopping the study if one or more participant experiences a serious adverse event attributable to IDA treatment. At each review, the DSMB will make a "go" ahead with the study or "no go" decision based on their analyses and assessment.

7.7 Procedures for Pausing the Trial

Upon review of the safety data from the trial, the DSMB will make decisions regarding the continuation of the trial. The DSMB will conduct an initial review after study drugs are distributed to approximately 10 participants in each treatment arm and consider halting or stopping the study if 1 or more participants in any treatment arm experiences an SAE by month 3 after treatment. The DSMB will pause to review data after any SAE then start again following the review and determination of the study's safety. Because participants will be recruited as cohorts, safety evaluations will be held on an ongoing basis and include cumulative safety data of grade 1 and 2 AEs and specific case information for grade 3 and above.

The DSMB has been convened to look out for the safety of study participants; it has the right and responsibility to pause the study if any of the treatments is associated with an unacceptable frequency of serious or severe adverse events. The DSMB should consult with the study medical monitor and PI before making a final decision to suspend the study. If the DSMB recommends discontinuation or modification of the study, the Chair of the DSMB will meet or speak to the DOLF Project Team at the earliest opportunity to review the basis for the recommendation, and the DOLF Project team will notify the Principal Investigator. The DSMB will begin data review meetings after enrollment of participants in the investigational arms of this study.
The DSMB and IRB/ERCs have the authority to stop the study at any point. The study may also be terminated by regulatory bodies if there is significant violation of GCP that compromises the ability to achieve the primary study objective or compromises subject safety.

However, as DSMB members are not on site, it is appropriate that they get input from the medical monitor and PI before making stopping or pausing decisions. The DSMB Chair will communicate the decision to discontinue or modify the study with the DOLF Project Team and the DOLF Project Team will immediately notify the PI of the DSMB's decision. The DOLF Team is in a better position to communicate with the DSMB Chair (only 1 hour time zone difference, more reliable internet, etc.).

8 STATISTICAL CONSIDERATIONS

8.1 Sample Size Determination (Power calculation)

The *primary outcome* variable used for our power analysis is the % fertile female worms (100 x fertile female worms/ total female worms) for each of the three drug treatments: (1) single-dose IVM (IVM alone); (2) single dose of IVM + DEC and albendazole (IDA1); (3) three daily doses of IVM + DEC and albendazole (IDA3). Power calculations for our primary treatment-group comparison (IVM /ALB vs IDA1) assumed that 30% of the total worms will be fertile females in IVM/ALB group and that 15% of the total worms will be fertile females in the IDA1 group. For our secondary treatment-group comparison (IVM/ALB vs IDA3), we assumed that 10% of the total worms were fertile females for the IDA3 group. On average, we assumed that there will be 6 total female worms to evaluate in 3 nodules per person after treatment. In our analysis we treated each worm within a subject as a Bernoulli trial (0=non-fertile female worm, 1=fertile female worm), and we used an intra-class correlation (ICC) of 30% to account for within subject correlation among the trials. The ICC and cluster size (# female worms/person) adjusts the sample-size estimate using the following formula: 1 + ICC x (Cluster size - 1) [44, 45]. To achieve 80% power at a two-sided alpha-level of 0.05, we will need nodule histology data from 51 subjects in each treatment group for the primary treatment-group comparison (IVM/ALB vs IDA1). The expected % difference for our secondary treatment-group comparison (IVM/ALB vs IDA3) powered at 80% and an alpha of 0.05, would require 26 subjects/group. After accounting for a 20% drop-out rate, a sample size of at least 61 subjects per treatment group will enable us to address both the primary (IVM/ALB vs IDA1) and secondary (IVM/ALB vs IDA3) treatment-group comparisons. Furthermore, we will attempt to enroll additional subjects beyond the target of 61 to provide some protection against other (unforeseen) factors that could decrease the power of this study (e.g., within village correlation, smaller effect size, higher drop-out rate than anticipated). The study will provide preliminary safety data, but a much larger study will be required to provide a precise safety comparison of IVM/ALB vs IDA.

8.2 Data Analysis Plan

Efficacy analysis for primary outcome

Because of the within subject correlation for the primary outcome (% fertile female worms), we will use a generalized linear mixed model approach (PROC GLIMMIX, SAS) [46]. Generalized linear mixed models (GLMM) permit modeling of non-normal outcomes while accounting for correlation among observations within a group. In the present study, the primary outcome is binary (0=non-fertile female worm, 1=fertile female worm) and worms within a person are not independent. To model these data we will use a GLMM assuming a binomial distribution with a logit link function,

and person will be treated as a random effect. Further, while we do not anticipate an effect of village on our outcomes, if subjects within village are correlated the GLMM can be extended to account for this correlation. A two-sided *P*-value ≤ 0.05 is considered significant for these analyses.

Efficacy analysis for secondary outcomes

Similar to the analysis of the primary outcome, percentage of all adult worms that are viable will be analyzed using a GLMM. Percent of persons with zero MF at 18 months will be analyzed using a generalized linear model assuming a binomial distribution and a logit link function. The difference between pre and post treatment MF counts (% reduction) will be analyzed using a paired t-test and will be transformed as necessary to meet the assumptions of this analysis. A *P*-value of 0.05 will be considered significant for all secondary outcomes.

Safety

The primary safety analysis will focus on AEs with severity grade 3 or greater. Secondary analyses will separately consider all AEs and ocular AEs. AEs will be assessed daily for 7 days after treatment and 3 months after treatment. This will include general assessments and detailed ophthalmological examinations on day 7 and 3 months after treatment. AE rates and severity will be compared across the three treatment groups using logistic regression analysis; with Student's *t*-test will be used to assess differences for individual AEs. The data will also be examined using a generalized linear mixed model that as described below can model non-normal outcomes and examine correlations among the different events and how these change over time. In this model, we can also examine the impact of baseline Mf burdens prior to treatment and other potential risk factors (age, sex, location, etc.) on AEs.

Note that the samples sizes per treatment group in this study are not sufficient to provide a robust safety profile (e.g., high confidence that the rate of SAEs after treatment is less than 0.1%). This proof of concept study will allow for initial safety assessments to be performed. Additional, larger studies will be required to establish a more complete safety profile. The safety assessments put in place for this study along with the frequent and ongoing review by the DSMB will provide a strong foundation to evaluate the participant's safety throughout the study.

Data from all participants in this study will be analyzed for safety and efficacy outcomes. The study population for the primary and secondary endpoints related to adult worm viability and fertility are persons who were enrolled and who have nodulectomies performed 18 months after treatment. The study population for secondary efficacy outcomes related to microfilariae in the skin are participants who were enrolled in the study who have follow-up microfilaria testing by skin snip examination at time points (12, 18 months) as indicated in the diagram on page 26 of the protocol. The study population for the early (7 day) safety analysis are participants who receive the study drugs who have safety assessments during the 7 days after dosing. A separate safety analysis will consider data from the follow-up examinations conducted 3 months after dosing.

8.3 Interim and Final Reports

An interim clinical trial report will be prepared and submitted to the Ghana FDA, IRB/ECs, and other relevant regulatory agencies after the 3-month safety assessments are complete. A final

report will be prepared and submitted to the same groups not later than 90 days after the completion of the trial (after the last patient, last visit for nodulectomy). Investigators will prepare these reports in accordance with the comprehensive formal report conforming to the *ICH E3 Guideline for the Structure and Content of Clinical Study Reports* as per sub-section 3.6.4.1 of the FDA's Guidelines for Authorization of Clinical Trials.

The biostatistician will generate tables, figures and listing during data analysis (after final follow-ups) during which time, they will review the data for missing, unused and spurious data. Study staff will report any major changes to the statistical analysis plan to the IRB at UHAS and the Ghana Health Service and to the DSMB. In addition, any major changes will be reported to the FDA prior to datalock.

9 DATA HANDLING

Data will be collected using a tablet-based system, pre-loaded with study templates. Study staff will be trained in the use of the instruments and data will be uploaded as entries are completed. Washington University in St. Louis will serve as the data coordinator for the study. The data will be owned by study investigators at UHAS School of Public Health Research Centre. The data will be compiled, validated and cleaned at UHAS School of Public Health Research Centre. Data will be accessible to Washington University in St. Louis for further validation checks and data cleaning. After data have been cleaned and finalized, the database will be locked, and the final dataset will be accessible to investigators at the University of Health and Allied Sciences School of Public Health Research Centre. Data analysis will be a collaborative effort between University of Health and Allied Sciences School of Public Health Research Centre and investigators at Case Western Reserve University and Washington University in St. Louis.

9.1 Types of Data Collected

Enrollment Data

- Participant identifier
- Informed consent date
- Demographic information (date of birth, gender)
- Pregnancy/breastfeeding status
- Medical history
- Presence of onchocercal nodules
- History of prior MDA treatment
- Physical exam
- Skin exam (including photographs of skin lesions)
- Ocular exam (including retinal and ocular coherence tomography photographs)

Laboratory Results

- Skin snip (including MF count)
- Nodule histology results (number of live/dead female and male worms, etc.)

Participant Monitoring and Follow-up

- Physical exam
- Skin exam (including photographs of skin lesions)
- Ocular exam (including retinal and ocular coherence tomography photographs)

Adverse Event Evaluation

- Adverse event assessment
- Treatment assignment
- Concomitant medication
- Ae description
- Start and stop date
- Outcome
- SAE evaluation and causality to MDA (definite, probable, possible, or unrelated)

9.2 Study Records Retention

Study documents will be retained for a minimum of seven (7) years after the study has been closed. These documents will be retained for a longer period if required by local regulations. No record will be destroyed without the written consent of DOLF and UHAS investigators.

The principle investigator at the study site will maintain appropriate medical and research records for this trial, in compliance with ICH E6, Section 4.9, regulatory and institutional requirements for the protection of confidentiality of participants. The investigator will permit authorized representatives of the sponsor and regulatory agencies to examine (and when required by applicable law, copy) clinical records for the purposes of clinical site monitoring, quality assurance reviews, audits, and evaluation of the study safety and progress.

9.3 Participant Key/Participant List

The participant key linking the list of all study participants with their unique identifier will only be available to the Ghanaian PI and to study personnel who have been delegated authority to handle the participant key. The participant key may be paper or electronic. The participant key will not be shared with investigators outside of Ghana.

9.4 Source Documents

This study may use paper and/or electronic source records. An electronic data capture system (EDC) will be employed to record data either directly into electronic forms or entered into the EDC from paper forms. Paper data collection forms derived from the electronic case report forms (eCRFs) will available to study staff as a backup. The selected EDC vendor will be 21 CFR 11 compliant including audit trails, time stamps, and encryption with secure password protected access.

Source documents will be stored for the duration of the study plus 7 years. The electronic database (study data) will be stored for 7 years by the Principle Investigators' institution. Source documents may be stored longer if required by regulatory requirements.

10 FUTURE USE OF STORED SPECIMENS

Samples collected in this study may be used for research related to onchocerciasis (diagnosis, parasite variability, treatment, or the pathogenesis of adverse events). Any other uses will require the prior approval of either the Washington University or University Hospitals Cleveland Medical Center (UHCMC) IRB, and the GHS-Ethical Review Committee and the UHAS Research Ethics Committee. Any use of samples outside of Ghana will require removal of any identifying information apart from study identification numbers. Subjects may still participate in this study even if they do not consent to use of their samples for future scientific studies. If the subject checks "no," (in the informed consent document), then their samples will be stripped of its identification number in the database after the completion of this study and the samples will not be used.

11 ETHICAL ISSUES

11.1 Informed Consent Process

Project investigators will consent eligible individuals in their own villages prior to the 6-month follow up of the Part I study. The Site PI or a co-investigator will inform the subjects of all aspects of the study covering all elements of informed consent. The study details will be explained in the local language Konkomba or Twi or in another language understood by the participant.

Project investigators will consent eligible individuals in their communities prior to enrollment into the IDA study. The Site PI or a co-investigator will inform the subjects of all aspects of the study covering all elements of informed consent. The study details will be read and explained in the local language (Konkomba or Twi) to the participant.

An informed consent document that is specific to the study will be available in English and the local language (Appendix F). The investigator must use an IRB/ERC approved informed consent document. Adequate time will then be allowed for the subjects to ask questions and make individual voluntary decisions. The form will be signed by the participant (or thumbprint) witnessed by an impartial community member and signed by an investigator (Dr. Opoku, Dr. Doe, Dr. Kanza, Dr. Gyasi). A copy of the information document and the consent will be given to the subject. A second copy of the consent will be stored in the Investigator's file. Only individuals who have signed the consent form and meet eligibility criteria will be enrolled in the study.

After the 18 month follow-up and nodulectomies, analysis will be done to determine the effectiveness of the drug therapies. All participants in less effective treatment arms will be offered the more effective treatment at no cost to the participant.

11.2 Comprehension of Informed Consent

To assess comprehension of informed consent and the study, the principal investigator and/or authorized consent study staff will ask the following questions:

1. Do you understand why you are being asked to participate?
2. Have you had the opportunity to ask questions and discuss the study?
3. Do you voluntarily participate in this study?

4. Are you willing to take part in all the study procedures?
5. Will we take blood from you during this study?
6. How long will you be in this study?
7. Do you understand you are free to stop being in the study at any time without having to give a reason?
8. Is there any charge for being in the study?
9. Do you understand that you will be compensated for lost wages?
10. Do you understand that you are participating in research and not receiving treatment or cure?
11. Do you know who to call if you have questions?

The responses will be documented on the consent form, signed by the research staff or investigators, and a copy given to the study volunteer. Participants will not be enrolled unless they can correctly answer each of these questions. In the event a subject indicates a lack of understanding of the study, or any aspect of it, the Principal Investigators/co-investigators/study staff will invite questions and offer explanations of any particular point. If, in the judgment of the Principal Investigators, the subject's response still does not reflect an understanding of the study, the subject will not be enrolled in the study.

11.3 Provisions for Subjects from Vulnerable Populations

Plan to avoid treating pregnant women.

Pregnant women will not be eligible to participate in this study because of the unknown effects of the investigational products in pregnant women. Women of child-bearing age who wish to enroll in the study will have a pregnancy test prior to enrolment in the study to ensure they are not pregnant. Lactating women who had a delivery more than 1 (one) month prior to potential enrollment can be enrolled/treated.

Plan for Inclusion of Illiterate Subjects

Study participants, if illiterate, will have the consent form read to them in the local languages (Konkomba, or Twi). Their signature or mark (thumbprint) will be witnessed on the consent form.

Plan for Inclusion of Non-English Speaking Individuals

Subjects who do not speak or read English are neither specifically included nor excluded from this study. Informed consent documents will be translated into Konkomba and Twi languages since all the participants enrolled into the ocular microfilarial clearance study speak one or both languages**. .**

Minors

Since minors can be affected by ocular onchocerciasis, participants aged 16-17 years will be included in this study. Younger children will not be included, because *O. volvulus* infections are generally less frequent and of lower intensity in that group and because younger children may not fully understand the potential risks and benefits of this first study of IDA in onchocerciasis.

If a minor (16 or 17 years of age) wishes to participant in the study, he/she and a parent/legal guardian must participate in the informed consent process. The informed assent form will be signed or thumb-printed by the minor, by at least one parent or legal guardian, by an independent witness, and by the principal investigator or his designee.

11.4 Alternatives to participation

Study subjects may decline participation in this study with no consequences. Subjects with known Onchocerciasis will be advised to join MDA with IVM available in their communities. .

Participants who are excluded from study due to severe eye disease or Mf in the posterior segment will be referred to the appropriate Ghana Health Service facility for management.

11.5 Withdrawal

Entry into the study and participation will be strictly voluntary. It will be made clear that refusal to participate or a decision to withdraw can occur at any time throughout the course of the study and will not influence their rights or the care they receive at local health facilities.

In two of the three treatment arms the participants receive a single treatment. In the 3rd treatment arm the patients receive treatment on three consecutive days. While there is no dose titration in place the investigator has the discretion to discontinue treatment if the treatment appears to be creating distress to the participant. The study investigator would monitor these participants closely and assess any adverse events that have occurred. There is no plan in place for the study investigator to re-initiate treatment once it has been discontinued.

Withdrawal from the study can be at the participant or investigator discretion at any time during the trial. All subjects that withdraw from the study will be encouraged to complete the safety follow-up visits (daily for 7 days after dosing and at 3 months).

Data collected on the participant up to the point of withdrawal remainspart of the study database. If the participant withdraws from the interventional part of the study and agrees to continue being seen for the safety follow-up visits the associated clinical outcome information such as medical information and laboratory results will be part of the study database.

There is no plan to replace withdrawn participants.

Protocol deviations are collected through the electronic data capture system (i.e. visits out of window, in-appropriate dosing, etc.) and reviewed through the tables, figures, listings. Additionally, a Protocol Deviation Log has been put in place to address deviations not picked up by EDC. Participants who fail to report for follow-up examinations will not be considered to have withdrawn, and the study team will attempt to contact them for follow-up testing at later time points listed in the protocol.

11.6 Participant Privacy

Privacy of the study participants will be maintained by assigning a unique study identification number (UNID). All data, skin snip samples and results will be recorded and analyzed by UNID

with no personal identifiers. All information collected, including name, specific address, etc. that could identify subjects will be kept confidential and available only to the investigators and authorized study personnel.

All written forms (i.e., consent and any paper data collection forms) will be stored in a designated locked area with limited access. All forms will be labelled and filed in cabinets with the study protocol number, PI's names and collection dates. These cabinets will be metal and have functioning locks. Keys will be kept with the study site PI, Data Manager, or Study Coordinator. Any electronic devices used to enter or collect data will be password protected. PIs and/or Project Coordinator will authorize access. The paper forms will be stored for the duration of the study plus seven years per IRB protocol for primary data storage.

Data will be entered into an electronic data capture system (EDC) that is 21 CFR 11 compliant. No participant names will be collected in the EDC. The database will be password protected and the Principal Investigators will authorize access to the password. For data transfer outside of the EDC data files will be exported as transfer files and, to ensure confidentiality, all files will be encrypted and password protected.

11.7 Plan for Subjects at the End of the Protocol

We will hold meetings at the end of the study in participating communities where the results of the study and significance of these results will be communicated to participants. At the end of the study participants will be informed to participate in MDA distributed in their communities. Results will also be shared with the Ghana Health Service.

12 RESPONSIBILITIES

12.1 Good Clinical Practice

The investigator will ensure that the basic principles of Good Clinical Practice are followed along with the appropriate laws and regulations of Ghana.

12.2 Clinical Trial Monitoring

An independent person or group experienced in monitoring clinical trials will be employed to help with clinical monitoring. This will include a pre-study GCP training and site assessment and site initiation monitoring. Additional monitoring will include evaluation of the study at one year post-drug administration, and a close out assessment following nodulectomies.

12.3 Institutional Review Board (IRB)/Ethics Review Committee (ERC)

This protocol will reviewed by the following IRB/ERCs:

- Research Ethics Committee of the University of Health and Allied Sciences (UHAS, Ho, Ghana)
- Institutional Review Board of the University Hospitals (Cleveland, USA)
- Washington University in St. Louis Institutional Review Board
- Ghana Health Service Ethics Review Committee
- Ghana Food and Drugs Authority (FDA)

The protocol and any accompanying material to be provided to the participants such as the informed consent will be submitted to the UHAS Research Ethics Committee, Ghana Health service Ethics Review Committee (GHS-ERC) and the Ghana FDA for review and approval. Approval from the committees must be obtained before starting the study and should be documented in correspondence to the investigator.

Any modifications to the protocol after receipt of the IRB or ERC approval must be submitted to the UHAS Research Ethics Committee and Ghana Health service Ethics Review Committee (GHS-ERC) and the Ghana FDA for approval prior to implementation.

12.4 Data Ownership

Data will be owned by UHAS. However, it is understood that UHAS will share study data with Principal Investigators, Co-investigators and key personnel for use in publications, presentations at scientific meetings or as preliminary data for subsequent grant applications. Confidentiality of study participants will be maintained by not using names or personal identifiers. The project will be subject to the DOLF Project Data Sharing and Publication Policy (Appendix G).

UHAS will permit access to all documents and records that may require inspection by the funding agencies, governmental regulatory agencies, institutional review boards (both UHCMC IRB and GHS-ERC) or its authorized representatives.

12.5 Financial Disclosure

The investigators listed in this protocol have no financial interests in the investigational products being researched in this study. This is an investigator-initiated study and the DOLF Project at Washington University in St. Louis Medical School is the sponsor of the study. The study is funded by a grant from the Bill and Melinda Gates Foundation. There is no financial agreement between the sponsor, funder, nor other investigators whereby the value of compensation would be influenced by the outcome of the study.

12.6 DOLF Team Technical Support

The DOLF Project Team conceived the idea of the study and obtained funding, DOLF developed the protocol with input from the P.I. DOLF provides technical support (e.g. data management, regulatory compliance support, statistical analysis, on-site training) and supplies for the conduct of the trial. The DOLF team will work with the P.I.s to analyze the data and publish results from the trial.

13 LITERATURE REFERENCES

1. Herricks JR, Hotez PJ, Wanga V, Coffeng LE, Haagsma JA, Basanez MG, Buckle G, Budke CM, Carabin H, Fevre EM, et al., 2017. The global burden of disease study 2013: What does it mean for the NTDs? PLoS Negl Trop Dis 11: e0005424.

2. Organization WH, 2016. Progress report on the elimination of human onchocerciasis, 2015–2016. Weekly epidemiological record 34: 501–516.
3. Taylor MJ, Awadzi K, Basanez MG, Biritwum N, Boakye D, Boatin B, Bockarie M, Churcher TS, Debrah A, Edwards G, et al., 2009. Onchocerciasis Control: Vision for the Future from a Ghanian perspective. Parasit Vectors 2: 7.
4. Zimmerman PA, Dadzie KY, De Sole G, Remme J, Alley ES, Unnasch TR, 1992. Onchocerca volvulus DNA probe classification correlates with epidemiologic patterns of blindness. J Infect Dis 165: 964-968.
5. Fischer P, Kipp W, Bamuhiga J, Binta-Kahwa J, Kiefer A, Buttner DW, 1993. Parasitological and clinical characterization of Simulium neavei-transmitted onchocerciasis in western Uganda. Trop Med Parasitol 44: 311-321.
6. Dadzie KY, Bird AC, Awadzi K, Schulz-Key H, Gilles HM, Aziz MA, 1987. Ocular findings in a double-blind study of ivermectin versus diethylcarbamazine versus placebo in the treatment of onchocerciasis. Br J Ophthalmol 71: 78-85.
7. Donnelly JJ, Taylor HR, Young E, Khatami M, Lok JB, Rockey JH, 1986. Experimental ocular onchocerciasis in cynomolgus monkeys. Invest Ophthalmol Vis Sci 27: 492-499.
8. Taylor HR, 1990. Onchocerciasis. Int Ophthalmol 14: 189-194.
9. Bird AC, el-Sheikh H, Anderson J, Fuglsang H, 1980. Changes in visual function and in the posterior segment of the eye during treatment of onchocerciasis with diethylcarbamazine citrate. Br J Ophthalmol 64: 191-200.
10. Duke BO, 1990. Human onchocerciasis--an overview of the disease. Acta Leiden 59: 9-24.
11. Braun G, McKechnie NM, Connor V, Gilbert CE, Engelbrecht F, Whitworth JA, Taylor DW, 1991. Immunological crossreactivity between a cloned antigen of Onchocerca volvulus and a component of the retinal pigment epithelium. J Exp Med 174: 169-177.
12. Chandrashekar R, Curtis KC, Weil GJ, 1995. Molecular characterization of a parasite antigen in sera from onchocerciasis patients that is immunologically cross-reactive with human keratin. J Infect Dis 171: 1586-1592.
13. Chandrashekar R, Ogunrinade AF, Alvarez RM, Kale OO, Weil GJ, 1990. Circulating immune complex-associated parasite antigens in human onchocerciasis. J Infect Dis 162: 1159-1164.
14. Greene BM, Gbakima AA, Albiez EJ, Taylor HR, 1985. Humoral and cellular immune responses to Onchocerca volvulus infection in humans. Rev Infect Dis 7: 789-795.
15. Johnson TP, Tyagi R, Lee PR, Lee MH, Johnson KR, Kowalak J, Elkahloun A, Medynets M, Hategan A, Kubofcik J, et al., 2017. Nodding syndrome may be an autoimmune reaction to the parasitic worm Onchocerca volvulus. Sci Transl Med 9.
16. Kawabata M, Izui S, Anan S, Kondo S, Fukumoto S, Flores GZ, Kobayakawa T, 1983. Circulating immune complexes and their possible relevance to other immunological parameters in Guatemalan onchocerciasis. Int Arch Allergy Appl Immunol 72: 128-133.
17. Semba RD, Murphy RP, Newland HS, Awadzi K, Greene BM, Taylor HR, 1990. Longitudinal study of lesions of the posterior segment in onchocerciasis. Ophthalmology 97: 1334-1341.
18. Banla M, Tchalim S, Karabou PK, Gantin RG, Agba AI, Kere-Banla A, Helling-Giese G, Heuschkel C, Schulz-Key H, Soboslay PT, 2014. Sustainable control of onchocerciasis: ocular pathology in onchocerciasis patients treated annually with ivermectin for 23 years: a cohort study. PLoS One 9: e98411.
19. Rodriguez-Perez MA, Fernandez-Santos NA, Orozco-Algarra ME, Rodriguez-Atanacio JA, Dominguez-Vazquez A, Rodriguez-Morales KB, Real-Najarro O, Prado-Velasco FG, Cupp EW, Richards FO, Jr., et al., 2015. Elimination of Onchocerciasis from Mexico. PLoS Negl Trop Dis 9: e0003922.

20. Diawara L, Traore MO, Badji A, Bissan Y, Doumbia K, Goita SF, Konate L, Mounkoro K, Sarr MD, Seck AF, et al., 2009. Feasibility of onchocerciasis elimination with ivermectin treatment in endemic foci in Africa: first evidence from studies in Mali and Senegal. PLoS Negl Trop Dis 3: e497.
21. Zarroug IM, Hashim K, ElMubark WA, Shumo ZA, Salih KA, ElNojomi NA, Awad HA, Aziz N, Katabarwa M, Hassan HK, et al., 2016. The First Confirmed Elimination of an Onchocerciasis Focus in Africa: Abu Hamed, Sudan. Am J Trop Med Hyg 95: 1037-1040.
22. Eisenbarth A, Achukwi MD, Renz A, 2016. Ongoing Transmission of Onchocerca volvulus after 25 Years of Annual Ivermectin Mass Treatments in the Vina du Nord River Valley, in North Cameroon. PLoS Negl Trop Dis 10: e0004392.
23. Katabarwa MN, Eyamba A, Nwane P, Enyong P, Kamgno J, Kuete T, Yaya S, Aboutou R, Mukenge L, Kafando C, et al., 2013. Fifteen years of annual mass treatment of onchocerciasis with ivermectin have not interrupted transmission in the west region of cameroon. J Parasitol Res 2013: 420928.
24. Katabarwa MN, Eyamba A, Nwane P, Enyong P, Yaya S, Baldiagai J, Madi TK, Yougouda A, Andze GO, Richards FO, 2011. Seventeen years of annual distribution of ivermectin has not interrupted onchocerciasis transmission in North Region, Cameroon. Am J Trop Med Hyg 85: 1041-1049.
25. Evans DS, Unnasch TR, Richards FO, 2015. Onchocerciasis and lymphatic filariasis elimination in Africa: it's about time. Lancet 385: 2151-2152.
26. Fischer PU, King CL, Jacobson JA, Weil GJ, 2017. Potential Value of Triple Drug Therapy with Ivermectin, Diethylcarbamazine, and Albendazole (IDA) to Accelerate Elimination of Lymphatic Filariasis and Onchocerciasis in Africa. PLoS Negl Trop Dis 11: e0005163.
27. Taylor HR, George T, 1987. Microfilaria in the cornea in onchocerciasis. Trans R Soc Trop Med Hyg 81: 148.
28. Greene BM, Taylor HR, Brown EJ, Humphrey RL, Lawley TJ, 1983. Ocular and systemic complications of diethylcarbamazine therapy for onchocerciasis: association with circulating immune complexes. J Infect Dis 147: 890-897.
29. Greene BM, Taylor HR, Cupp EW, Murphy RP, White AT, Aziz MA, Schulz-Key H, D'Anna SA, Newland HS, Goldschmidt LP, et al., 1985. Comparison of ivermectin and diethylcarbamazine in the treatment of onchocerciasis. N Engl J Med 313: 133-138.
30. Basanez MG, Pion SD, Boakes E, Filipe JA, Churcher TS, Boussinesq M, 2008. Effect of single-dose ivermectin on Onchocerca volvulus: a systematic review and meta-analysis. Lancet Infect Dis 8: 310-322.
31. Taylor HR, Murphy RP, Newland HS, White AT, D'Anna SA, Keyvan-Larijani E, Aziz MA, Cupp EW, Greene BM, 1986. Treatment of onchocerciasis. The ocular effects of ivermectin and diethylcarbamazine. Arch Ophthalmol 104: 863-870.
32. Awadzi K, Opoku NO, Attah SK, Lazdins-Helds J, Kuesel AC, 2014. A randomized, single-ascending-dose, ivermectin-controlled, double-blind study of moxidectin in Onchocerca volvulus infection. PLoS Negl Trop Dis 8: e2953.
33. Taylor HR, 1990. Ivermectin treatment of ocular onchocerciasis. Acta Leiden 59: 201-206.
34. Taylor HR, Semba RD, Newland HS, Keyvan-Larijani E, White A, Dukuly Z, Greene BM, 1989. Ivermectin treatment of patients with severe ocular onchocerciasis. Am J Trop Med Hyg 40: 494-500.
35. Thomsen EK, Sanuku N, Baea M, Satofan S, Maki E, Lombore B, Schmidt MS, Siba PM, Weil GJ, Kazura JW, et al., 2016. Efficacy, Safety, and Pharmacokinetics of Coadministered

Diethylcarbamazine, Albendazole, and Ivermectin for Treatment of Bancroftian Filariasis. Clin Infect Dis 62: 334-341.
36. WHO, 2017. Guideline - alternative mass drug administration regimens to eliminate lymphatic filariasis. King JD, ed, 1-71.
37. Merck, 2017. Merck Commemorates 30 Years of MECTIZAN® Donation Program Progress: Program Expanded to Reach Additional 100 Million People Annually for Lymphatic Filariasis in Support of New, Evidence-Based WHO Guidelines. Available at: http://investors.merck.com/news/press-release-details/2017/Merck-Commemorates-30-Years-of-MECTIZAN-Donation-Program-Progress/default.aspx. Accessed August 10, 2018, 2018.
38. Awadzi K, Gilles HM, 1992. Diethylcarbamazine in the treatment of patients with onchocerciasis. Br J Clin Pharmacol 34: 281-288.
39. Opoku NO, Bakajika DK, Kanza EM, Howard H, Mambandu GL, Nyathirombo A, Nigo MM, Kasonia K, Masembe SL, Mumbere M, et al., 2018. Single dose moxidectin versus ivermectin for Onchocerca volvulus infection in Ghana, Liberia, and the Democratic Republic of the Congo: a randomised, controlled, double-blind phase 3 trial. Lancet.
40. Bird AC, Anderson J, Fuglsang H, 1976. Morphology of posterior segment lesions of the eye in patients with onchocerciasis. Br J Ophthalmol 60: 2-20.
41. Fuglsang H, Anderson J, 1978. Further observations on the relationship between ocular onchocerciasis and the head nodule, and on the possible benefit of nodulectomy. Br J Ophthalmol 62: 445-449.
42. Wojtkowski M, Bajraszewski T, Gorczynska I, Targowski P, Kowalczyk A, Wasilewski W, Radzewicz C, 2004. Ophthalmic imaging by spectral optical coherence tomography. Am J Ophthalmol 138: 412-419.
43. Jolodar A, Fischer P, Buttner DW, Miller DJ, Schmetz C, Brattig NW, 2004. Onchocerca volvulus: expression and immunolocalization of a nematode cathepsin D-like lysosomal aspartic protease. Exp Parasitol 107: 145-156.
44. Donner A, Koval JJ, 1981. A multivariate analysis of family data. Am J Epidemiol 114: 149-154.
45. Rutterford C, Copas A, Eldridge S, 2015. Methods for sample size determination in cluster randomized trials. Int J Epidemiol 44: 1051-1067.
46. Littel RC, Milliken GA, Stroup WW, Wolfinger RD, Schabenberger O, 2006. SAS for mixed models. North Carolina: SAS Institute Inc.

14 APPENDICES

APPENDIX A: Sample Participant ID Card

APPENDIX B: Investigational Product Package Inserts

APPENDIX C: Sample Adverse Event Assessment Forms

APPENDIX D: CTCAE Scoring Tables

APPENDIX E: DSMB Charter

APPENDIX F: Informed Consent Form

APPENDIX G: Data Sharing Agreement
