# Supplementary figures and images for: A randomized, open-label study of the tolerability and efficacy of one or three daily doses of ivermectin plus diethylcarbamazine and albendazole (IDA) versus one dose of ivermectin plus albendazole (IA) for treatment of onchocerciasis
Source: PLoS Negl Trop Dis. 2023 May 19;17(5):e0011365. doi: 10.1371/journal.pntd.0011365 (PMC10234528; doi:10.1371/journal.pntd.0011365)

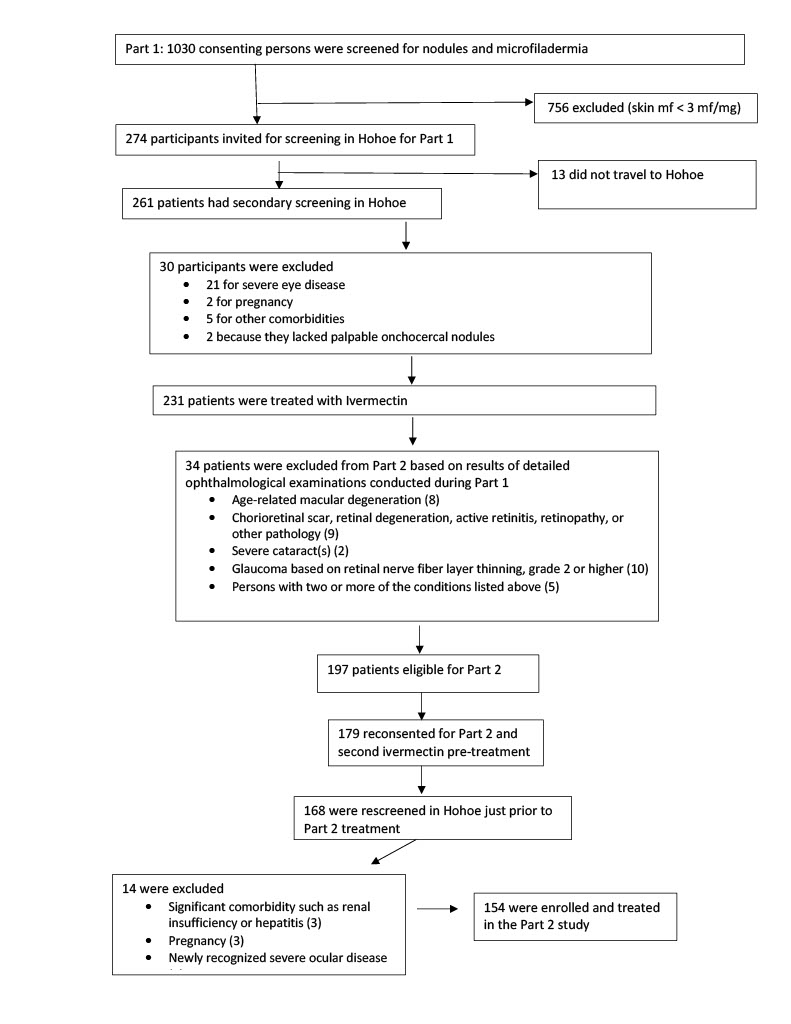

Supplement: S1 Fig — (JPG) [file pntd.0011365.s002.jpg]
