# Supplementary material for: A randomized, open-label study of the tolerability and efficacy of one or three daily doses of ivermectin plus diethylcarbamazine and albendazole (IDA) versus one dose of ivermectin plus albendazole (IA) for treatment of onchocerciasis
Source: PLoS Negl Trop Dis. 2023 May 19;17(5):e0011365. doi: 10.1371/journal.pntd.0011365 (PMC10234528; doi:10.1371/journal.pntd.0011365)
Supplement: S1 Table — (DOCX) [file pntd.0011365.s003.docx]

**Table S1. Ocular adverse events (AE) following treatment.**

| Subject | MfAC | Treatment | Adverse event | AE grade | Mazzotti Reaction | Time post treatment | Resolved by 3 mo | Change in pinhole Visual Acuity logMAR |
| --- | --- | --- | --- | --- | --- | --- | --- | --- |
| GV1044 | 0 | IA | Cataract | mild | No | Month 3 | No | 0 |
| GV1057 | 0 | IA | Itching | mild | No | Day 3 | Yes | 0 |
| GV1075 | 0 | IA | Eye Pain | mild | No | Day 1 | Yes | 0 |
|  |  |  | Tearing | No data | No | Day 1 | Data n/a | 0 |
|  |  |  | Itching | mild | No | Day 3 | Yes | 0 |
|  |  |  | Vitritis | mild | No | Day 3 | Yes | 0 |
| GV1102 | 0 | IA | Itching | mild | No | Day 3 | Yes | 0 |
| GV1150 | 0 | IA | Itching | mild | No | Day 3 | Yes | -0.176 OD |
| GV1242 | 0 | IA | Cataract | mild | No | Month 3 | No | 0 |
| GV1033 | 0 | IDA1 | Itching | mild | No | Month 3 | Yes | 0 |
|  |  |  | Eye pain | mild | No | Month 3 | Yes | 0 |
| GV1078 | 0 | IDA1 | Vitritis | mild | No | Day 7 | Yes | 0 |
| GV1127 | 0 | IDA1 | Foreign body sensation | mild | No | Day 3 | Yes | 0 |
| GV1141 | 0 | IDA1 | Vitritis | mild | No | Day 3 | Yes | 0 |
| GV1187 | 0 | IDA1 | Retinal vascular disorder | mild | No | Day 3 | Yes | 0 |
| GV1244 | 0 | IDA1 | Itching | mild | No | Day 7 | Yes | +0.176 OD |
| GV1028 | 0 | IDA3 | Retinal vascular disorder | mild | No | Month 3 | No | 0 |
| GV1038 | 0 | IDA3 | Cataract | mild | No | Month 3 | No | -0.125 OU |
| GV1054 | 0 | IDA3 | Punctate Opacities | mild | No | Day 3 | No | 0 |
| GV1250 | 0 | IDA3 | Itching | moderate | Yes | Day 7 | Yes | 0 |
|  |  |  | Eye pain | mild | No | Month 3 | Yes | 0 |

Abbreviations: MfAC, (pretreatment microfilaria in the anterior chamber); IA, ivermectin plus albendazole; IDA1, single dose ivermectin plus diethylcarbamazine and albendazole; IDA3, three daily doses of IDA; OD, right eye; OU, both eyes.
