# Supplementary material for: A randomized, open-label study of the tolerability and efficacy of one or three daily doses of ivermectin plus diethylcarbamazine and albendazole (IDA) versus one dose of ivermectin plus albendazole (IA) for treatment of onchocerciasis
Source: PLoS Negl Trop Dis. 2023 May 19;17(5):e0011365. doi: 10.1371/journal.pntd.0011365 (PMC10234528; doi:10.1371/journal.pntd.0011365)
Supplement: S2 Table — (DOCX) [file pntd.0011365.s004.docx]

**Table S2** Additional histology results regarding embryogenesis in female *Onchocerca volvulus* worms present in nodules surgically excised 18 months after treatment.

|  | **IA (1X)** | **IDA (1X)** | **IDA3 (3X)** |
| --- | --- | --- | --- |
| Number of living female worms | 127 | 142 | 159 |
| Number of females without an evaluable uterus (%) | 17 (13.4) | 12 (8.5) | 20 (12.6) |
| Number if females with an evaluable uterus (%) | 110 (86.6) | 130 (91.5) | 139 (87.4) |
| Number of females with empty uterus (%) | 9 (8.2) | 14 (10.8) | 21 (15.1) |
| Number of females with only oocytes in the uterus (%) | 50 (45.5) | 66 (50.8) | 75 (54) |
| Number of females with degenerated, morulae, coiled, stretched microfilariae in the uterus (%) | 10 (9.1) | 10 (7.7) | 9 (6.5) |
| Number of females without normal embryogenesis (not producing) (%) | 69 (62.7) | 90 (69.2) | 105 (75.5) |
| Number of females with normal embryogenesis (morulae, coiled, or stretched microfilariae) (%) | 41 (37.3) | 40 (30.8) | 34 (24.5) |
